# Autotaxin inhibition attenuates the aortic valve calcification by suppressing inflammation-driven fibro-calcific remodeling of valvular interstitial cells

**DOI:** 10.1186/s12916-024-03342-x

**Published:** 2024-03-14

**Authors:** Dohee Yoon, Bongkun Choi, Ji-Eun Kim, Eun-Young Kim, Soo-Hyun Chung, Hyo-Jin Min, Yoolim Sung, Eun-Ju Chang, Jae-Kwan Song

**Affiliations:** 1 Department of Biochemistry and Molecular Biology, Brain Korea 21 Project, Asan Medical Center, University of Ulsan College of Medicine, 88 Olympic-ro 43-gil, Songpa-gu, Seoul, 05505 Republic of Korea; 2 Stem Cell Immunomodulation Research Center, Asan Medical Center, University of Ulsan College of Medicine, 88 Olympic-ro 43-gil, Songpa-gu, Seoul, 05505 Republic of Korea; 3 Division of Cardiology, Department of Internal Medicine, Asan Medical Center, University of Ulsan College of Medicine, 88 Olympic-ro 43-gil, Songpa-gu, Seoul, 05505 Republic of Korea

**Keywords:** Fibro-calcific aortic valve disease, Autotaxin, Aortic valve, Fibrosis, Calcification

## Abstract

**Background:**

Patients with fibro-calcific aortic valve disease (FCAVD) have lipid depositions in their aortic valve that engender a proinflammatory impetus toward fibrosis and calcification and ultimately valve leaflet stenosis. Although the lipoprotein(a)-autotaxin (ATX)-lysophosphatidic acid axis has been suggested as a potential therapeutic target to prevent the development of FCAVD, supportive evidence using ATX inhibitors is lacking. We here evaluated the therapeutic potency of an ATX inhibitor to attenuate valvular calcification in the FCAVD animal models.

**Methods:**

ATX level and activity in healthy participants and patients with FCAVD were analyzed using a bioinformatics approach using the Gene Expression Omnibus datasets, enzyme-linked immunosorbent assay (ELISA), immunohistochemistry, and western blotting. To evaluate the efficacy of ATX inhibitor, interleukin-1 receptor antagonist-deficient (*Il1rn*^-/-)^ mice and cholesterol-enriched diet-induced rabbits were used as the FCAVD models, and primary human valvular interstitial cells (VICs) from patients with calcification were employed.

**Results:**

The global gene expression profiles of the aortic valve tissue of patients with severe FCAVD demonstrated that ATX gene expression was significantly upregulated and correlated with lipid retention (*r* = 0.96) or fibro-calcific remodeling-related genes (*r* = 0.77) in comparison to age-matched non-FCAVD controls. Orally available ATX inhibitor, BBT-877, markedly ameliorated the osteogenic differentiation and further mineralization of primary human VICs in vitro. Additionally, ATX inhibition significantly attenuated fibrosis-related factors’ production, with a detectable reduction of osteogenesis-related factors, in human VICs. Mechanistically, ATX inhibitor prohibited fibrotic changes in human VICs via both canonical and non-canonical TGF-β signaling, and subsequent induction of CTGF, a key factor in tissue fibrosis. In the in vivo FCAVD model system, ATX inhibitor exposure markedly reduced calcific lesion formation in interleukin-1 receptor antagonist-deficient mice (*Il1rn*^-/-^, *P* = 0.0210). This inhibition ameliorated the rate of change in the aortic valve area (*P* = 0.0287) and mean pressure gradient (*P* = 0.0249) in the FCAVD rabbit model. Moreover, transaortic maximal velocity (*V*max) was diminished with ATX inhibitor administration (mean *V*max = 1.082) compared to vehicle control (mean *V*max = 1.508, *P* = 0.0221). Importantly, ATX inhibitor administration suppressed the effects of a high-cholesterol diet and vitamin D2-driven fibrosis, in association with a reduction in macrophage infiltration and calcific deposition, in the aortic valves of this rabbit model.

**Conclusions:**

ATX inhibition attenuates the development of FCAVD while protecting against fibrosis and calcification in VICs, suggesting the potential of using ATX inhibitors to treat FCAVD.

**Supplementary Information:**

The online version contains supplementary material available at 10.1186/s12916-024-03342-x.

## Background

In this era of aging societies, aortic stenosis (AS) has become the most prevalent cardiac disease. Prodigious advances in percutaneous interventional approaches have contributed to optimal treatment options for AS, but despite the effectiveness of either surgical or transcatheter valve replacements, only obstructions due to end-stage disease are amenable to treatment. The clinical importance of primary or secondary lifestyle modifications and medical therapies to prevent the initiation or progression of AS cannot be overemphasized [1, 2]. Although the term “degenerative AS” has been used, implying an inevitable, ineluctably progressive, and inherent process, the demonstration that valvular endothelial cells (VECs) and valvular interstitial cells (VICs) maintain valvular homeostasis and structural integrity has rendered a paradigm shift in our understanding of the pathogenesis of AS [1]. Hence, the aortic valve is now considered to be “a metabolically active tissue” and not just an inactive or passive flap, and it is generally believed that, starting from inflammatory changes, the progression to fibrosis and calcification results in the development and progression of AS [3]. Thus, “fibro-calcific aortic valve disease (FCAVD)” has been suggested to be a more logical and scientific term than “degenerative AS” [4, 5].

FCAVD consecutively develops as initial endothelial dysfunction, inflammation, fibrotic change, and successive calcification of VICs [4]. Many risk factors, such as diabetes [6], hypertension, and aging [7], can promote endothelial dysfunction of VECs, leading to lipoprotein and immune cell infiltration [8]. Accumulated lipids are oxidized due to the dysregulation of the endothelial nitric oxide synthase (eNOS) pathway, and oxidized lipids also enhance immune cell extravasation [9]. Both oxidized lipids and infiltration of immune cells trigger inflammatory responses to produce inflammatory cytokines (e.g., IL-1β, TNF-α, and IL-6) that stimulate the transdifferentiation of VICs [10], leading to the pathogenic changes of the aortic valve [11–13]. These lipid depositions and chronic inflammation thus initiate FCAVD progression with subsequent fibrosis and calcification [1, 12].

Autotaxin (ATX) is a secreted lysophospholipase D that hydrolyzes extracellular lysophosphatidylcholine into the lipid mediator—lysophosphatidic acid (LPA), which is a ligand for specific G protein-coupled receptors [14]. In particular, since the expression of ATX is upregulated in the calcified aortic valve tissue [12], it is believed that ATX-LPA signaling is implicated in the great diversity of FCAVD pathophysiological processes. A previous study has confirmed that LPA promotes mineralization of aortic valve by inducing an osteogenic program in VICs [12, 14]. In addition, the LPA produced by ATX acts on mineralization within the aortic valves supporting that ATX-LPA can be a suitable target to prevent the progression of FCAVD [12]. However, the mechanism underlying the osteogenic effects driven by ATX-LPA signaling axis remains unclear. Although ATX inhibitors are in an active developmental stage for targeting idiopathic pulmonary fibrosis [15], their impact on the fibro-calcific transition of FCAVD has not yet been seriously investigated. In the present study, we evaluated molecular mechanisms by which inhibition of ATX can contribute to preventing the progression of FCAVD. In particular, we aimed to demonstrate that ATX inhibition provides dual beneficial effects preventing both fibrosis and calcification, which can be a unique advantage.

## Methods

### Human participants

Human aortic valve tissues and serums were obtained from patients with FCAVD who underwent aortic valve replacement. The research protocol for this study was approved by the Institutional Review Board of Asan Medical Center (Seoul, Republic of Korea), and all patients provided informed consent for the use of their clinical specimens for research (No. 2017-0556 for tissues and No. 2019-0965 for serums). Human VICs were prepared from the calcified human aortic valve specimens and cultured as previously described [16]. Briefly, the valve tissues were rinsed in Dulbecco’s modified Eagle’s medium (DMEM; Cytiva, Marlborough, MA, USA) and collagenase type I was used to remove valvular endothelial cells. To isolate VICs, the remaining leaflets were incubated with collagenase type I, collagenase type II, and dispase in DMEM at 37℃ and 5% CO_2_ for 2 h under constant shaking. Cells were collected by centrifugation and resuspended in DMEM growth medium supplemented with 20% fetal bovine serum (FBS; Cytiva), 100 units/mL penicillin (Cytiva), and 100 μg/mL streptomycin (Cytiva). Isolated VICs were cultured at 37℃ under 5% CO_2_ and used in passages 4 to 7 in the experiments. To induce the osteogenic differentiation of primary human VICs, the culture media was replaced with osteogenic media (DMEM suppled with 10% FBS, 10 nM dexamethasone, 10 mM β-glycerophosphate, and 0.25 mM L-ascorbic acid). The osteogenic media were changed every 2–3 days, and the cells were cultured for up to 3 weeks. This osteogenic transdifferentiation of VICs was evaluated by alkaline phosphatase (ALP) and alizarin red (AR) staining. After 2 weeks of osteogenic stimulation with osteogenic media, ALP staining was performed using an Alkaline Phosphatase Kit (Sigma-Aldrich, St. Louis, MO, USA) in accordance with the manufacturer’s protocol. For AR staining, cells were washed with PBS, fixed for 30 min in 4% paraformaldehyde, and exposed to 2% alizarin red solution for 10 min after 3 weeks of osteogenic stimulation. Calcium deposition was colorimetrically quantified in 0.1 M HCL extracts of cultured VICs, utilizing the o-cresolphthalein method, and spectrophotometrically analyzed at 450 nm. BBT-877 (kindly provided by Bridge Biotherapeutics, Gyeunggi, Republic of Korea), an orally available small molecule inhibitor of ATX, was used in both in vitro and in vivo experiments [17].

### Autotaxin activity assay

Autotaxin activity in conditioned media was measured using a commercial assay kit (Echelon Biosciences, Salt Lake City, UT, USA) in accordance with the manufacturer’s instructions.

HPLC for LPA analysis was performed through LegoChem Biosciences (Daejeon, Republic of Korea).

### Western blotting

For western blotting analysis, equal amounts of total protein prepared from cultured VICs were resolved by sodium dodecyl sulfate-polyacrylamide gel electrophoresis (SDS-PAGE) and electrophoretically transferred to a polyvinylidene difluoride (PVDF) membrane. Non-specific interactions were blocked using Protein-Based Blocking Reagent (Thermo Fisher Scientific, Waltham, MA, USA) for 1 h. After blocking, membranes were incubated with primary antibodies against p-Smad2, p-Smad3, Smad2/3, p-p38, p38, p-AKT (threonine 308), AKT, p-ERK, ERK, p-JNK, JNK (Cell Signaling Technology, Danvers, MA, USA), autotaxin (Abcam, Cambridge, UK), and GAPDH (Genetex, Irvine, CA, USA) overnight at 4℃. Membranes were then incubated with the secondary HRP-conjugated antibodies and detected using ECL western blotting detection reagent. The images of protein bands were quantified using ImageJ analysis software.

### Quantitative real-time polymerase chain reaction (qPCR)

Total RNA extracts were isolated from cultured VICs using QIAzol lysis reagent (Qiagen, Venlo, Netherlands) in accordance with the manufacturer’s protocols. These RNA preparations were then converted to cDNA with the RevertAid First Strand cDNA Synthesis kit (Thermo Fisher Scientific, Waltham, MA, USA). The synthesized cDNA was subsequently analyzed using a SYBR green real-time PCR using LightCycler 480 thermocycler (Roche, Basel, Switzerland) in accordance with the manufacturer’s instructions. The relative expression of the target genes was normalized using the GAPDH level.

### Enzyme-linked immunosorbent assay (ELISA)

Conditioned media obtained from VICs were measured using commercial enzyme-linked immunosorbent assay (ELISA) kits in accordance with the manufacturer’s protocols for transforming growth factor beta (TGF-β; R&D Systems, Minneapolis, MN, USA), connective tissue growth factor (CTGF; Abcam, Cambridge, UK), osteopontin (Abcam), and lysophosphatidic acid (LPA; MyBioSource, San Diego, CA, USA). For macrophage migration inhibitory factor (MIF), monocyte chemoattractant protein-1 (MCP-1), macrophage inflammatory protein-1 alpha (MIP-1α), macrophage inflammatory protein-1 beta (MIP-1β), C-X-C motif chemokine ligand 9 (MIG), C-X-C motif chemokine ligand 10 (IP-10), macrophage-colony stimulating factor (M-CSF), vascular endothelial growth factor (VEGF), insulin-like growth factor binding protein 3 (IGFBP-3), granulocyte-macrophage colony-stimulating factor (GM-CSF), thrombopoietin, FMS-like tyrosine kinase 3 ligand (FLT3L), tumor necrosis factor alpha (TNF-α), interferon gamma (IFN-γ), interleukin 1 alpha (IL-1α), interleukin 1 beta (IL-1β), interleukin 8 (IL-8), interleukin 12 (IL-12), interleukin 4 (IL-4), interleukin 10 (IL-10), TNF-related apoptosis-inducing ligand (TRAIL), interleukin 6 (IL-6), platelet-derived growth factor AA (PDGF-AA), platelet-derived growth factor BB (PDGF-BB), and basic fibroblast growth factor (bFGF), multiplex ELISA were performed through LABISKOMA company (Seoul, Republic of Korea) using human premixed multi-analyte kit. All samples were examined in triplicate for each experiment.

### Animal experiments

All animal experiments were conducted in compliance with the protocols which are based on the previous experiment results approved by the Institutional Committee for the Use and Care of Laboratory animals of Ulsan University (No. 2022-12-183) and following the ARRIVE Essential 10 guidelines. Interleukin-1 receptor antagonist-deficient (*Il1rn*^-/-^) mice in BALB/C background distinguished by genotyping generated by Horai et al. [18] were used as an animal model of FCAVD and compared with aged-matched littermate wild-type (WT). To minimize confounder, we conducted experiments through randomized administration of ATX inhibitor and randomized allocation of feeding-cage. Four-week-old female *Il1rn*^-/-^ mice were randomly assigned for orally administered saline or BBT-877, an orally viable small molecule inhibitor of ATX, at 30 mg/kg/day diluted in deionized water for 8 weeks. Mice were maintained on a 12-h light/dark cycle. At 8 weeks after saline or BBT-877 administration, molecular imaging of calcification was performed using a fluorescent bisphosphonate-conjugated imaging agent (Osteosense 680EX; VisEn Medical, Bedford, MA, USA). The experimental mice were injected via the tail vein with Osteosense 680EX and osteogenic activity was measured at 18 h. Osteosense 680EX binds to hydroxyapatite (calcified regions) and can identify osteoblast function and calcium deposition in vivo. After anesthetizing the mice with inhalation of isoflurane, calcific depositions were imaged using an IVIS spectrum in vivo imaging system (PerkinElmer, Shelton, CT, USA).

Four-week-old male New Zealand white rabbits were divided into three groups based on dietary intake and ATX inhibitor administration and observed for 12 weeks as follows: (1) normal chow (control group); (2) 0.5% cholesterol-enriched chow (Dyets, Bethlehem, PA, USA) with 25,000 IU/day vitamin D2 (VitD, Santa Cruz) (Chol+VitD group); and (3) Chol+VitD regimen with BBT-877 (1 mg/kg/day) orally for 12 weeks (Chol+VitD+BBT group). After 12 weeks, echocardiography was performed, and the rabbits were euthanized to collect their aortic valve tissues. All animal protocols were performed in accordance with the Institutional Committee for the Use and Care of Laboratory animals of Ulsan University guidelines (No. 2021-12-047). Echocardiographic examinations were performed in a rabbit FCAVD model after the animals had been anesthetized using an intramuscular injection of ketamine (30 mg/kg) and xylazine (6 mg/kg). The aortic valve area (AVA), transaortic maximal velocity (*V*max), and mean pressure gradient (mean PG) were measured as described previously. Briefly, The AVA was determined using the standard continuity equation. The left ventricular outflow tract (LVOT) diameter was measured in the parasternal long-axis view. The stroke volume measured with a pulsed-wave Doppler proximal to the aortic valve was assessed in an apical five-chamber view, as is regularly done in human patients. To estimate the integral of the transvalvular flow, a continuous wave Doppler was obtained for use in the continuity equation for AVA calculations and to record the *V*max and mean PG.

### Histological analysis

Aortic valve tissues were fixed in 4% paraformaldehyde, embedded in paraffin, sectioned serially at 4 μm, deparaffinized, and stained with hematoxylin and eosin (H&E; Abcam) for histological evaluation. The deparaffinized tissues were exposed to 2% alizarin red solution to detect mineralization. For immunohistochemical (IHC) assays, paraffin sections were incubated with autotaxin (Abcam, Cambridge, UK) or α-smooth muscle actin (α-SMA; Abcam) antibodies and developed using the Dako REAL EnVision Detection System Peroxidase/DAB+ (Dako, Glostrup, Denmark), in accordance with the manufacturer’s guidelines. For Masson’s trichrome staining, rehydrated paraffin sections were incubated in Bouin’s solution for fixation and were followed by staining with Weigert’s hematoxylin solution, Biebrich scarlet acid fusion solution, and aniline blue. Tissues were photographed with an Olympus SLIDEVIEW VS200 microscope (Olympus, Tokyo, Japan).

### Statistical analysis

All quantitative experiments were performed at a minimum of three times independently and the data were presented as a mean ± SD (standard deviation). Data analyses were conducted using GraphPad Prism 8 Software (https://www.graphpad.com). Data satisfying normality distribution were conducted a two-tailed *t*-test which was used to assess statistical significance. *P* values <0.05 was considered to indicate statistical significance (*P < 0.05, **P < 0.01, ***P < 0.001).

## Results

### Fibro-calcific gene expression changes associated with autotaxin in patients with FCAVD

**Fig. 1:**
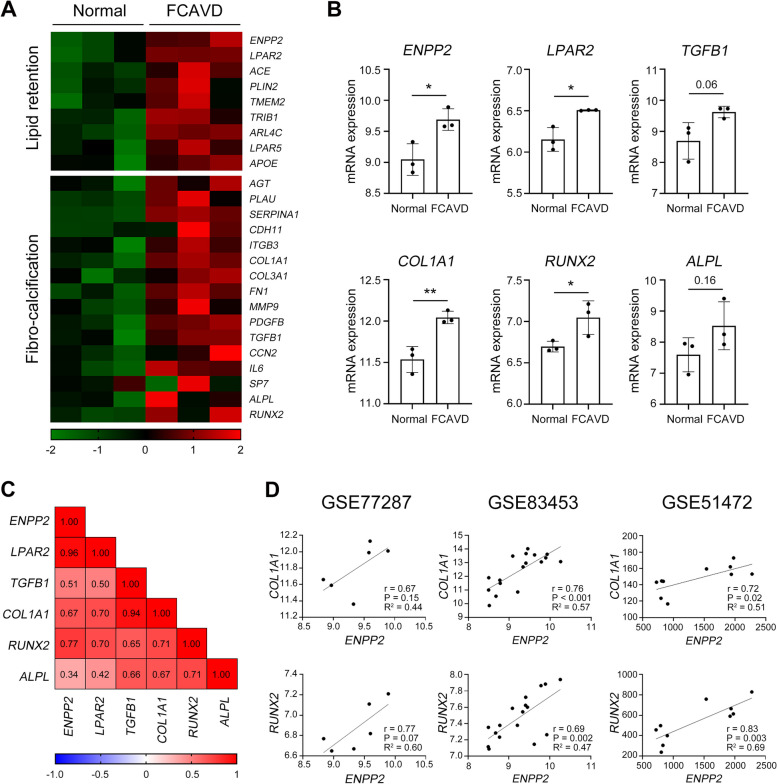
The fibro-calcific remodeling in patients with FCAVD is associated with the ATX-LPA axis.** A–D** Related genes of lipid retention and fibro-calcific changes were compared between healthy (*n* = 3) and FCAVD patients (*n* = 3). **A** Data were obtained from the GSE77287 dataset and visualized using MeV 4.9.0 heatmap software. **B** The mRNA expression of genes associated with lipid retention and fibro-calcific changes was compared between aortic valvular tissue of patients with FCAVD and age-matched non-FCAVD controls using a two-tailed *t*-test. **C** Pearson correlation values were further analyzed to examine the correlation between ATX (*ENPP2*) and selected fibro-calcific-related genes with up/up gene pairs exhibiting predominantly positive correlation. **D** Pearson’s correlation coefficient and linear regression array analysis of the correlation between the enhanced *ENPP2* and *COL1A1*, as well as *RUNX2* from GSE77287, GSE83453, and GSE51472 datasets. *P < 0.05, **P < 0.01 versus healthy sample. *P* values were obtained using a two-tailed *t*-test

Given that autotaxin (ATX) activity and LPA levels were upregulated in FCAVD samples compared to normal samples (Additional file 1: Fig. S1), we reasoned that lipid deposition and further LPA conversion would be correlated with the fibro-calcific remodeling in the aortic valve of FCAVD patients. Using the analysis of the global gene expression profiles (GEO) of our previously published dataset GSE77287 [16], the expression of lipid retention-related or fibro-calcific remodeling-related genes in the aortic valve tissues of patients with severe FCAVD and age-matched non-FCAVD control was determined (Fig. 1A). ATX (*ENPP2*), LPA receptor 2 (*LPAR2*), the fibrotic factor TGF-β (*TGFB1*), collagen type 1 α 1 chain (*COL1A1*), runt-related transcription factor 2 (*RUNX2*), and alkaline phosphatase (*ALPL*) expressions were specifically upregulated in the aortic valve tissue of patients with FCAVD compared to those in the aortic valve tissue control (Fig. 1B). To determine the contribution of ATX on the fibro-calcific process in the aortic valve, Pearson correlation analyses were performed between expression levels of these genes and ATX expressions. The genes related to fibro-calcific pathways were positively correlated with ATX expression using data from GSE77287 (Fig. 1C). Positive correlations were observed for *COL1A1* and *RUNX2* (*COL1A1*: *r* = 0.67, *RUNX2*: *r* = 0.77) in accordance with the previous other public datasets GSE51472 (*COL1A1*: *r* = 0.72, *RUNX2*: *r* = 0.83) [19] and GSE83453 (*COL1A1*: *r* = 0.76, *RUNX2*: *r* = 0.69) [20] (Fig. 1D and Additional file 1: Fig. S2). These data showed a close relationship between ATX expression and fibro-calcific remodeling in the aortic valve of patients with FCAVD.

### Attenuation of in vitro osteogenic transition in human VICs by the ATX inhibitor

**Fig. 2:**
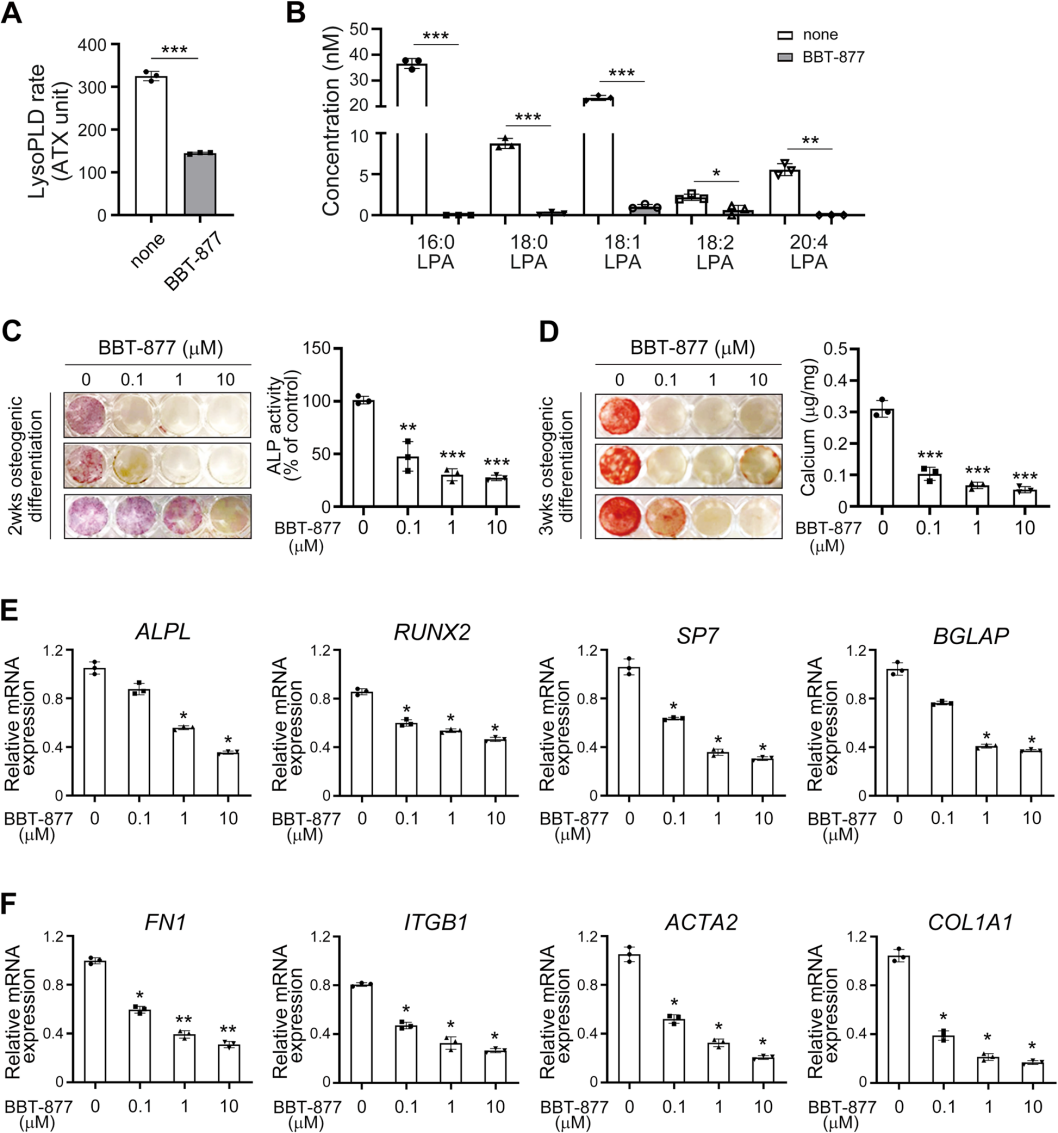
Attenuation of osteogenic transition and osteogenesis-related gene expression in human VICs cultured from FCAVD patients by treatment with ATX inhibitor. **A–F** Human VICs were cultured in osteogenic differentiation conditions in the presence of BBT-877 or vehicle control. Effects of BBT-877 treatment (1 μM) on ATX activity (**A**) and LPA species production (**B**) from VICs during osteogenic differentiation were measured as described in the “Methods” section. **C** Alkaline phosphatase (ALP) staining and ALP activity of these cells upon exposure to the indicated concentrations of BBT-877 (0, 0.1, 1, 10 μM) after 2 weeks of osteogenic stimulation. **D** Alizarin red (AR) staining and calcium deposition of the VICs in the presence (0.1, 1, 10 μM) or absence of BBT-877 after 3 weeks of osteogenic stimulation. **E** The mRNA expression levels of *ALPL*, *RUNX2*, *SP7*, and *BGLAP* in the VICs in the presence (0.1, 1, 10 μM) or absence of BBT-877. **F** The mRNA expression levels of *FN1*, *ITGB1*, *ACTA2*, and *COL1A1* in VICs in the presence (0.1, 1, 10 μM) or absence of BBT-877. Data are presented as the mean ± SD of triplicates, and a representative set of findings from more than three independent experiments is presented. *P < 0.05, **P < 0.01, ***P < 0.001 versus vehicle control. *P* values were obtained using a two-tailed *t*-test

BBT-877 is an edible ATX inhibitor established in a murine model of pulmonary fibrosis [17]. We first examined the inhibitory effects of BBT-877 on ATX activity in osteogenic cultures of VICs. The activity of ATX in the culture medium of VICs was significantly reduced upon treatment with 1 μM of BBT-877, without any observed cytotoxic effects on cells (Fig. 2A and Additional file 1: Fig. S3). In addition, the production of LPA species (LPA 16:0, LPA 18:0, LPA 18:1, LPA18:2, and LPA 20:4), which are products converted by ATX [21], was almost completely inhibited by BBT-877 (Fig. 2B). Since BBT-877 was found in our current experiments to suppress LPA production during the osteogenic differentiation of VICs in vitro, we further determined whether it could affect the osteogenic changes in these cells. BBT-877 treatment led to a significant reduction in calcification of VICs, as revealed by ALP staining, which coincided with a decrease in ALP activity (Fig. 2C). Moreover, the BBT-877 treatment of VICs notably attenuated matrix mineralization, as revealed by alizarin red staining after 3 weeks of stimulation (Fig. 2D, left). This diminution corresponded to a parallel decrease in the calcium concentrations in the culture medium (Fig. 2D, right). We next evaluated whether BBT-877 attenuated the expression of osteogenesis-related genes in VICs. A 1-μM dose was found to reduce the mRNA expression levels of *ALPL*, *RUNX2*, Sp7 transcription factor (*SP7*, also known as Osterix), and bone γ-carboxyglutamic acid-containing protein (*BGLAP*, also known as osteocalcin) (Fig. 2E). These reductions were greater at a 10 μM BBT-877 concentration, confirming that ATX inhibitor can effectively reduce the osteogenic transition of VICs. Furthermore, BBT-877 significantly attenuated the expression of fibrosis-related genes including fibronectin 1 (*FN1*), integrin subunit β 1 (*ITGB1*), smooth muscle α-2 actin (known as α-SMA and encoded by *ACTA2* gene), and collagen type 1 α 1 chain (encoded by *COL1A1*) (Fig. 2F).

### ATX inhibition downregulates both osteogenesis and fibrosis-related cytokines

**Fig. 3:**
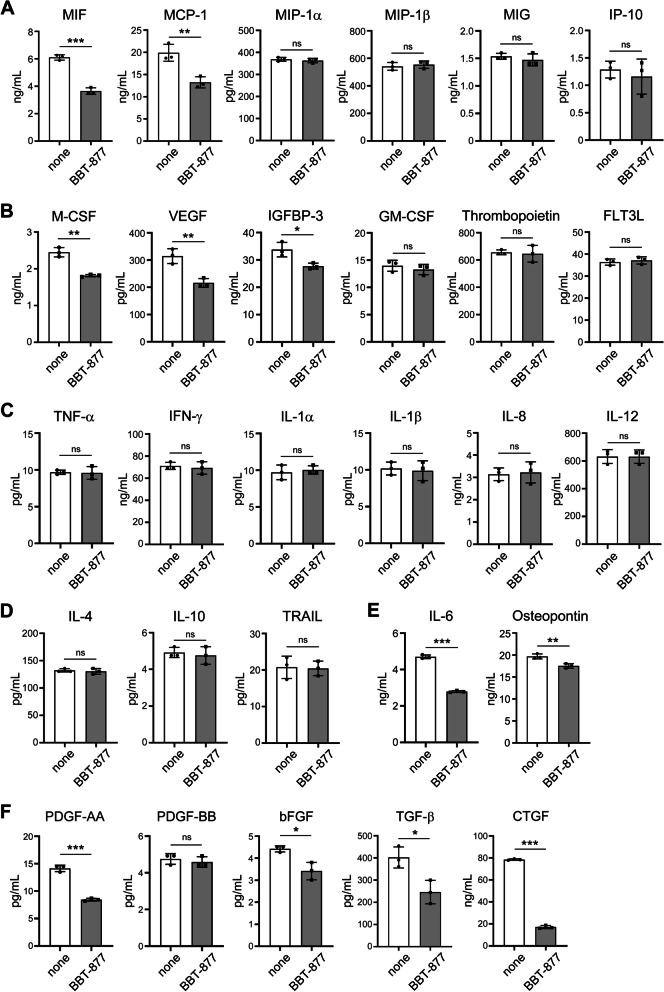
Effects of ATX inhibition on the production of cytokines related to the infiltration of immune cells, proinflammatory/anti-inflammatory responses, and fibrosis and osteogenesis in human VICs from FCAVD patients. **A–F** The human VICs were cultured in an osteogenic medium for 3 weeks in the presence or absence of BBT-877 (1 μM), and the indicated cytokines were measured by ELISA. **A** Chemokines. **B** Growth factors for immune cells. **C** Proinflammatory cytokines. **D** Anti-inflammatory cytokines. **E** Cytokines related to osteogenesis. **F** Cytokines related to fibrosis. Data are presented as the mean ± SD of triplicate ELISA tests. A representative set of results from more than three independent experiments is presented. *P < 0.05, **P < 0.01, ***P < 0.001 versus vehicle control. *P* values were obtained using a two-tailed *t*-test

Previous studies have reported that FCAVD develops through the following sequential cellular events: endothelial dysfunction, lipid deposition, infiltration of immune cells and induction of inflammation, fibrotic changes, and the subsequent calcification of VICs [1, 22]. Following endothelial dysfunction, both VECs and VICs are reported to produce cytokines to induce immune cell infiltration and the maturation/activation of infiltrated cells [23, 24]. To define whether ATX inhibition can directly affect cellular events in VICs that promote the pathogenesis of FCAVD, we thus validated the profiling of the production of chemokines and growth factors by VICs using multiplex protein assay in the presence or absence of BBT-877 after 3 weeks of osteogenic stimulation (Fig. 3). Among the chemokines we measured, MIF and MCP-1, which induce the infiltration of myeloid cells, were downregulated by exposure to BBT-877 (Fig. 3A). Additionally, M-CSF, which facilitates the maturation of infiltrated myeloid cells, and VEGF, which induces angiogenesis, were decreased by ATX inhibition of the cells (Fig. 3B). Interestingly, IGFBP-3, which we previously found to exacerbate the onset of FCAVD by downregulating IGF-1 signaling [16], was also decreased by BBT-877 treatment (Fig. 3B). We additionally measured cytokines that would further affect inflammation but none of the proinflammatory cytokines such as TNF-α, IFN-γ, or IL-1β (Fig. 3C), or anti-inflammatory cytokines such as IL-4, IL-10, or TRAIL (Fig. 3D), were affected by BBT-877. This inhibition thus affects inflammatory events through the induction of immune cell infiltration, rather than via the secretion of pro/anti-inflammatory cytokines, in VICs. Hence, ATX activity inhibition is suggested by our current findings to attenuate the progression of FCAVD by downregulating the infiltration of immune cells in the initial stages of FCAVD development. With regard to further osteogenesis and calcification, the IL-6 and osteopontin levels in media were significantly reduced by BBT-877 treatment (Fig. 3E). Next, cytokines known to induce the fibrosis and calcification of VICs [1, 4, 25] were analyzed. PDGF-AA and bFGF, which are important for the induction of fibrosis, were decreased in VICs BBT-877 treatment, although the level of PDGF-BB remained unchanged (Fig. 3F). Importantly, TGF-β, a master regulator of osteoinduction and fibrotic changes [1, 4] was significantly decreased during osteogenic differentiation when the cells were exposed to this inhibitor. CTGF, encoded by the *CCN2* gene, is expressed downstream of TGF-β and directly affects fibrosis [26]. The expression level of CTGF was also markedly reduced by BBT-877 treatment. Collectively, these results revealed therapeutic effects of ATX inhibition on the fibrosis and osteogenic differentiation of VICs, and partial effects in terms of downregulating immune cell infiltration, that attenuate the development of FCAVD.

### ATX inhibition attenuates the osteogenic differentiation of VICs by targeting the TGF-β signaling pathway

**Fig. 4:**
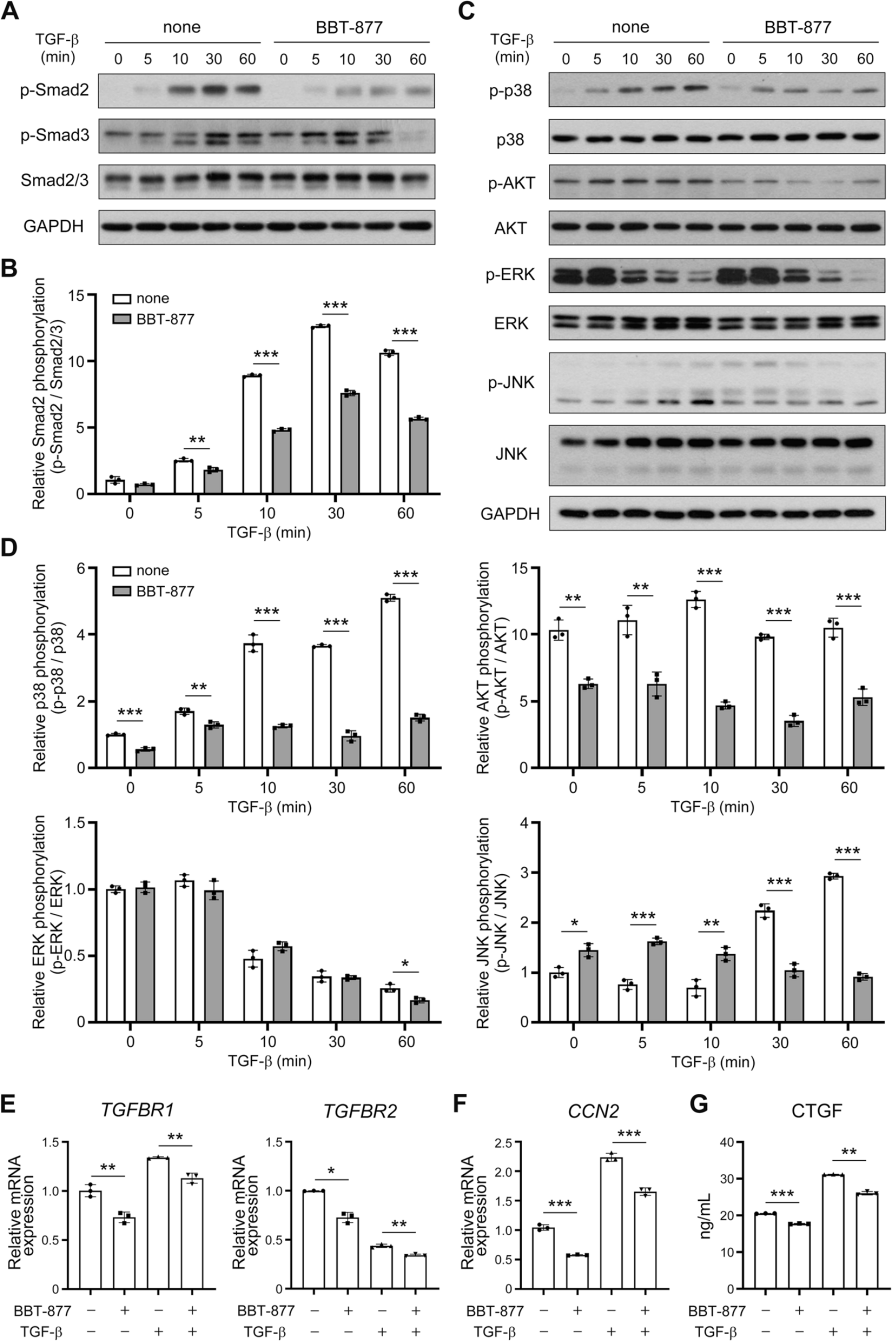
ATX inhibition of human VICs isolated from FCAVD patients causes the downregulation of the TGF-β pathway and related fibrosis processes. **A** The protein levels of p-Smad2, p-Smad3, Smad2/3, and GAPDH in VICs treated with TGF-β (5 ng/mL) for 0, 5, 10, 30, or 60 min in the presence or absence of BBT-877 (1 μM). **B** Quantification of phospho-Smad2 levels relative to the total Smad2/3 concentration. Immunoblots were quantified using ImageJ software. **C**, **D** Protein levels of p-p38, p38, p-AKT, AKT, p-ERK, ERK, p-JNK, and JNK in VICs treated with TGF-β (5 ng/mL) for 0, 5, 10, 30, or 60 min in the presence or absence of BBT-877 (1 μM). **E**, **F** mRNA expression levels of TGF-β receptor 1 (*TGFBR1*), TGF-β receptor 2 (*TGFBR2*), and cellular communication network factor 2 (CTGF, *CCN2*) in VICs after 24 h of TGF-β (5 ng/mL) stimulation in the presence or absence of BBT-877 (1 μM). **G** CTGF in VICs-conditioned media after 24 h of TGF-β (5 ng/mL) stimulation in the presence or absence of BBT-877 (1 μM). Data are presented as the mean ± SD and the experiments were performed independently in triplicate. **P < 0.01, ***P < 0.001 versus the vehicle control. *P* values were obtained using a two-tailed *t*-test

We next confirmed whether the regulatory effects on osteogenic differentiation of VICs through ATX inhibitor occurred via the downstream signaling pathways of TGF-β, which involve the basic Smad signaling pathway, and non-canonical pathways that transfer signals through other signaling molecules [27]. In our first experiment, the Smad pathway was examined during TGF-β stimulation of VICs by western blotting (Fig. 4A). BBT-877 treatment downregulated the phosphorylation of Smad2 at 5, 10, 30, and 60 min after exposure to TGF-β (Fig. 4B). Phosphorylation of Smad3 was also decreased after 30 and 60 min of TGF-β stimulation. Moreover, consistent with the effects observed on the Smad pathway of the TGF-β, phosphorylation of p38, AKT, ERK, and JNK were also downregulated in the treated cells (Fig. 4C, D). These data indicated that ATX inhibition hinders both canonical and non-canonical TGF-β signaling. Subsequently, we confirmed that BBT-877 suppressed the transcription of TGF-β receptors (Fig. 4E). The mRNA expression levels of both TGF-β receptors, *TGFBR1* and *TGFBR2*, were decreased by BBT-877 treatment regardless of the presence or absence of TGF-β stimulation. To determine the potential impacts of ATX inhibition on fibrosis through the regulation of CTGF production, we measured its mRNA and protein expression levels in the treated cells (Fig. 4F, G). As expected, BBT-877-treated VICs expressed much lower basal levels of *CCN2* transcripts than the vehicle control cultures, and an equivalent reduction was seen after TGF-β exposure (Fig. 4F). The protein expression of CTGF in the conditioned media induced by TGF-β was also decreased in accordance with BBT-877 treatment (Fig. 4G), which indicates that ATX regulates fibrosis in VICs via the TGF-β signaling pathways. In addition, TGF-β augmented osteogenesis-related gene expressions such as *ALPL, SPP1, RUNX2, IL6,* and *BMP2* (bone morphogenetic protein 2) and these upregulations were diminished through BBT-877 treatment in VICs (Additional file 1: Fig. S4A). BBT-877 treatment also downregulated the increased IL-6 protein expression with TGF-β treatment in VICs-conditioned media (Additional file 1: Fig. S4B). These data suggest that the osteogenic changes in VICs occur concomitantly with fibrosis mediated by TGF-β and they can be suppressed by ATX inhibition.

### ATX inhibition suppresses in vivo calcification in the FCAVD animal models

**Fig. 5:**
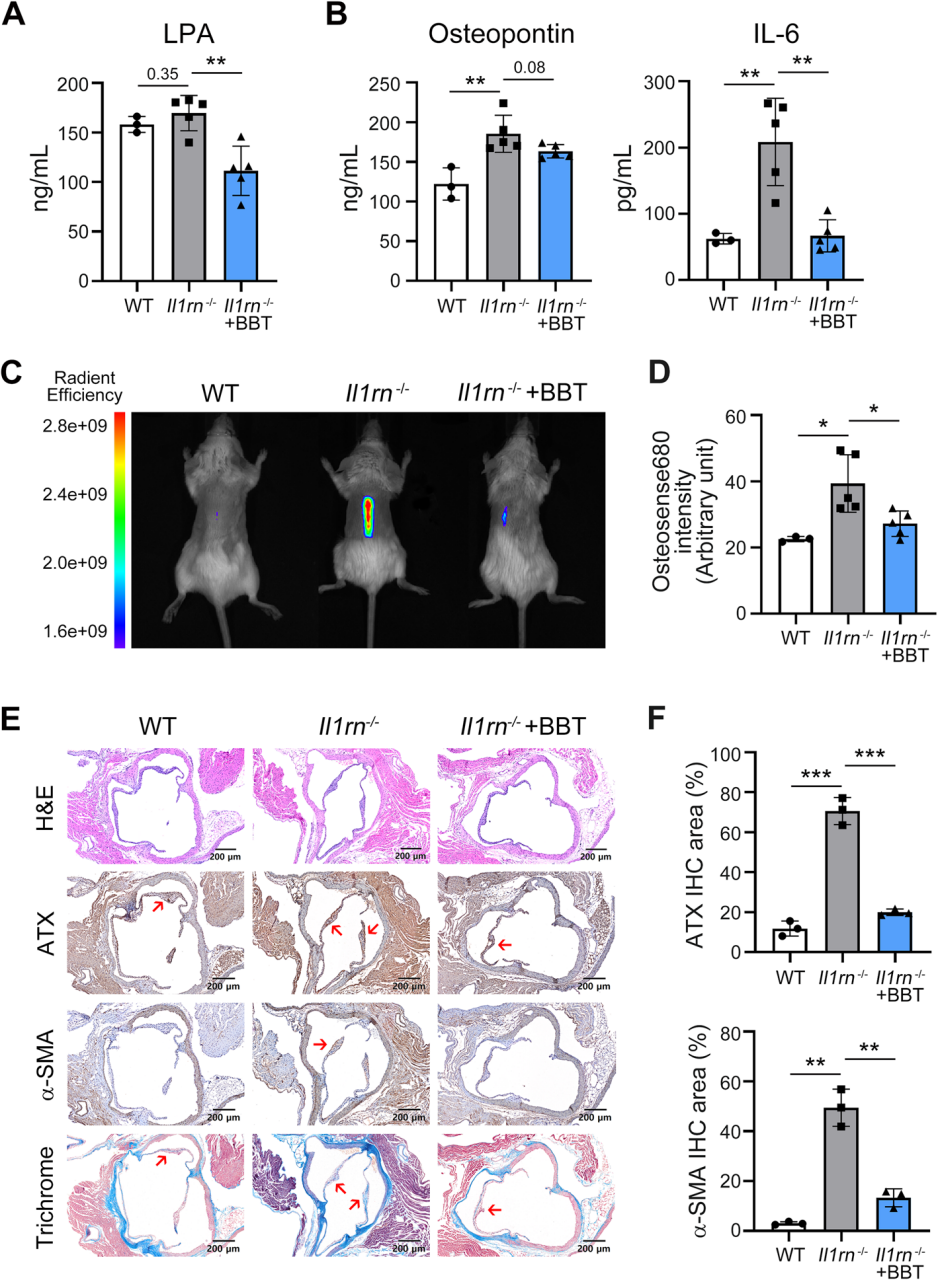
Alleviation of calcific lesion formation in a mouse FCAVD model, *Il1rn*^-/-^ mice by treatment with the ATX inhibitor. **A–F** *Il1rn*^-/-^ mice were orally administrated with BBT-877 (30 mg/kg/day) or vehicle for 8 weeks. **A, B** LPA production (**A**) and osteogenesis-related cytokines (**B**) in mouse serum were measured using ELISA. **C, D** Calcific lesion formation was determined using molecular imaging after injection of a fluorescence dye for calcification (Osteosense 680EX). Representative images of mice from each group (**C**) and quantification of fluorescence intensity (**D**) in all of the mice in each group. **E, F** Paraffin-embedded serial cross sections of the aortic valves from mice stained with hematoxylin and eosin (H&E) and analyzed by immunohistochemistry (IHC) and Masson’s trichrome staining (red arrow indicates stained positive area in the aortic valve). Antibodies against ATX and α-SMA were used for IHC analyses. Data are presented as the mean ± SD. *P < 0.05, ** *P* < 0.01, *** *P* < 0.001 versus the indicated group. *P* values were obtained using a two-tailed *t*-test. WT (*n* = 3); *Il1rn*^-/-^ (*n* = 5); *Il1rn*^-/-^+BBT-877 (*n* = 5)

It is well known that numerous inflammatory cytokines are involved in the progression of FCAVD, including TNF-α, IL-6, and IL-1β [11–13]. We confirmed that inflammatory stimuli could enhance the ATX-LPA signaling pathway in human VICs in vitro. Notably, IL-1β treatment upregulated LPAR1 and LPA expressions more effectively than TNF-α treatment (Additional file 1: Fig. S5). Interleukin 1 receptor antagonist-deficient (*Il1rn*^-/-^) mouse is one of the animal models of FCAVD with increased aortic valve leaflet thickness and fibrosis [28]. Therefore, we confirmed the therapeutic effects of ATX inhibition in *Il1rn*^-/-^ mice to verify the role of ATX in the development of FCAVD in vivo. We verified the decreased serum LPA level in BBT-877 administrated mice (WT: 158.0 ± 6.5, *Il1rn*^-/-^: 169.3 ± 7.2, *Il1rn*^-/-^+BBT: 111.2 ± 17.6, Fig 5A). Increased serum osteopontin and IL-6 expressions were diminished with BBT-877 administration (Fig. 5B). Molecular imaging of an injected fluorescent agent (Osteosense 680EX) revealed that a 30 mg/kg/day dosage of this inhibitor significantly suppressed calcification in vivo in the *Il1rn*^-/-^ mice compared to the vehicle control (WT: 22.5±0.6, *Il1rn*^-/-^: 39.3±6.5, *Il1rn*^-/-^+BBT: 27.2±2.9, Fig. 5C, D). Moreover, increased aortic valve thickness was diminished with ATX inhibition in *Il1rn*^-/-^ mice (Fig. 5E). Immunohistochemistry (IHC) analysis confirmed that ATX expression was decreased by BBT-877 (Fig. 5E, F). In addition, the vascular smooth muscle cell marker of fibrosis, α-SMA, and tissue fibrosis revealed by Masson’s trichrome staining were reduced by BBT-877 treatment. These data indicate that inhibition of ATX alleviates FCAVD in *Il1rn*^-/-^ mice.

**Fig. 6:**
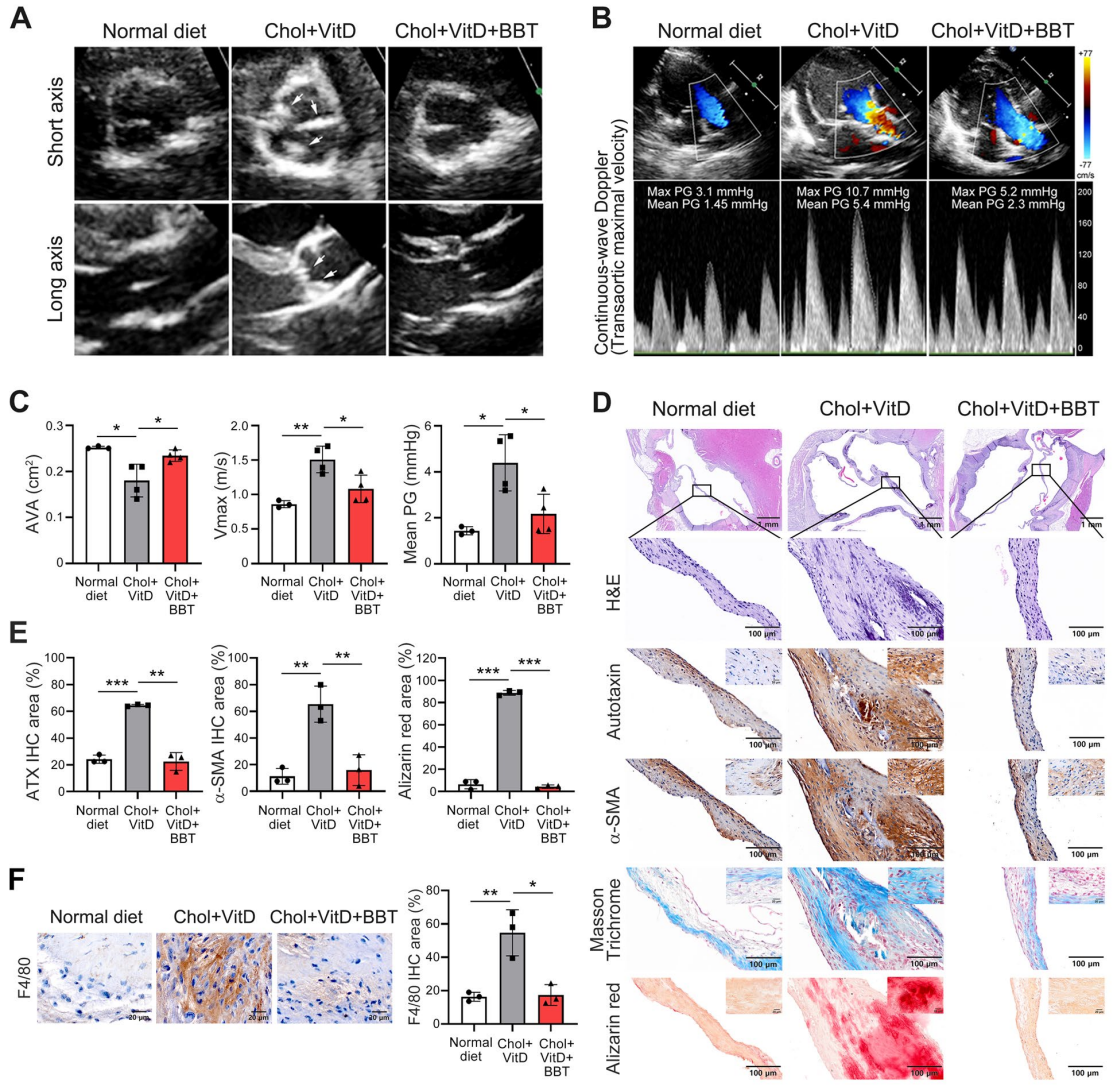
Attenuation of fibrosis and calcification by treatment with the ATX inhibitor in a rabbit model of FCAVD.** A** Representative 2D echocardiographic images of the aortic valve cusps in the rabbits at 12 weeks after BBT-877 oral administration. **B** Standard continuity equation of the aortic valve area (AVA). **C** AVA, transaortic maximal velocity (*V*max) and mean pressure gradient (mean PG) obtained by continuous-wave Doppler analysis through echocardiographic assessment. **D–F** Paraffin-embedded serial cross sections of the aortic valves from rabbits stained with hematoxylin and eosin (H&E), alizarin red, and analyzed by immunohistochemistry (IHC) and Masson’s trichrome staining. Antibodies against ATX, α-SMA, and F4/80 were used for IHC analyses. Normal diet (*n* = 3); Chol+VitD, (cholesterol and vitamin D2, disease group, *n* = 4); Chol+VitD+BBT, (cholesterol and vitamin D2 with BBT-877, BBT-877 group, *n* = 4). Data are presented as the mean ± SD. *P < 0.05, **P < 0.01, ***P < 0.001 versus the vehicle control. *P* values were obtained using a two-tailed *t*-test

Because of the anatomical differences between mouse and human aortic valves, rabbit models are considered to be a more accurate system for researching the pathogenesis of FCAVD due to greater similarities in the human and rabbit tri-layer valvular morphologies [29]. We thus generated an FCAVD model in a New Zealand white rabbit (NZW) background by administrating high cholesterol diet (Chol) and vitamin D2 (VitD) as described previously [30] and examined the therapeutic effects of ATX inhibition on calcification in these animals. The rabbits were randomly divided into three groups on the basis of their diets, i.e., (1) normal diet (normal group); (2) cholesterol with vitamin D2 supplementation (Chol+VitD, disease group); and (3) BBT-877 group with Chol+VitD (Chol+VitD+BBT). Although the aortic valve cusps were thickened after 12 weeks of treatment, the administration of BBT-877 notably maintained a similar cusp morphology to the control group (Fig. 6A). Next, the transvalvular flow was measured using continuous wave Doppler to calculate the aortic valve area (AVA; Fig. 6B). The AVA, transaortic maximal velocity (*V*max), and mean pressure gradient (mean PG) were then measured to quantify the effects of ATX inhibition (AVA; normal diet: 0.25 ± 0.0023, Chol+VitD: 0.18 ± 0.029, Chol+VitD+BBT: 0.23 ± 0.0083) (Fig. 6C). The disease group (Chol+VitD) displayed a significant decrease in the AVA, as measured using a standard continuity equation, while the BBT-877-treated animals (Chol+VitD+BBT) showed a similar level to that observed in the control group. The *V*max and mean PG were relatively increased in the disease group due to a narrowed aortic valve but were comparable between the BBT-877 and normal groups. All of the Doppler analysis data thus indicated that ATX inhibition attenuates the progression of arterial stenosis in the rabbit model of FCAVD.

After performing the aforementioned echocardiographic assessments, we isolated the aortic valves from the FCAVD rabbits to analyze histological changes (Fig. 6D, E). H&E staining demonstrated a marked thickening of the aortic valve cusps in the disease group compared to the normal diet group. However, the cusps from the BBT-877-treated group were as thin as those of the normal control group. Immunohistochemical analysis revealed that enhanced ATX and α-SMA expressions in the aortic valve were decreased with BBT-877 administration. Consistent with the previous results, this treatment mitigated the tissue fibrosis, as indicated by Masson’s trichrome staining. We further found that calcium deposits, revealed by alizarin red staining, were markedly diminished in the BBT-877-treated group, compared with the disease group. In addition, the reduction in F4/80 staining indicated that macrophage infiltration was mitigated by BBT-877 treatment (Fig. 6F). These results clearly validate the therapeutic effects of ATX inhibition against FCAVD in vivo through the attenuation of fibrosis and calcification.

## Discussion

**Fig. 7:**
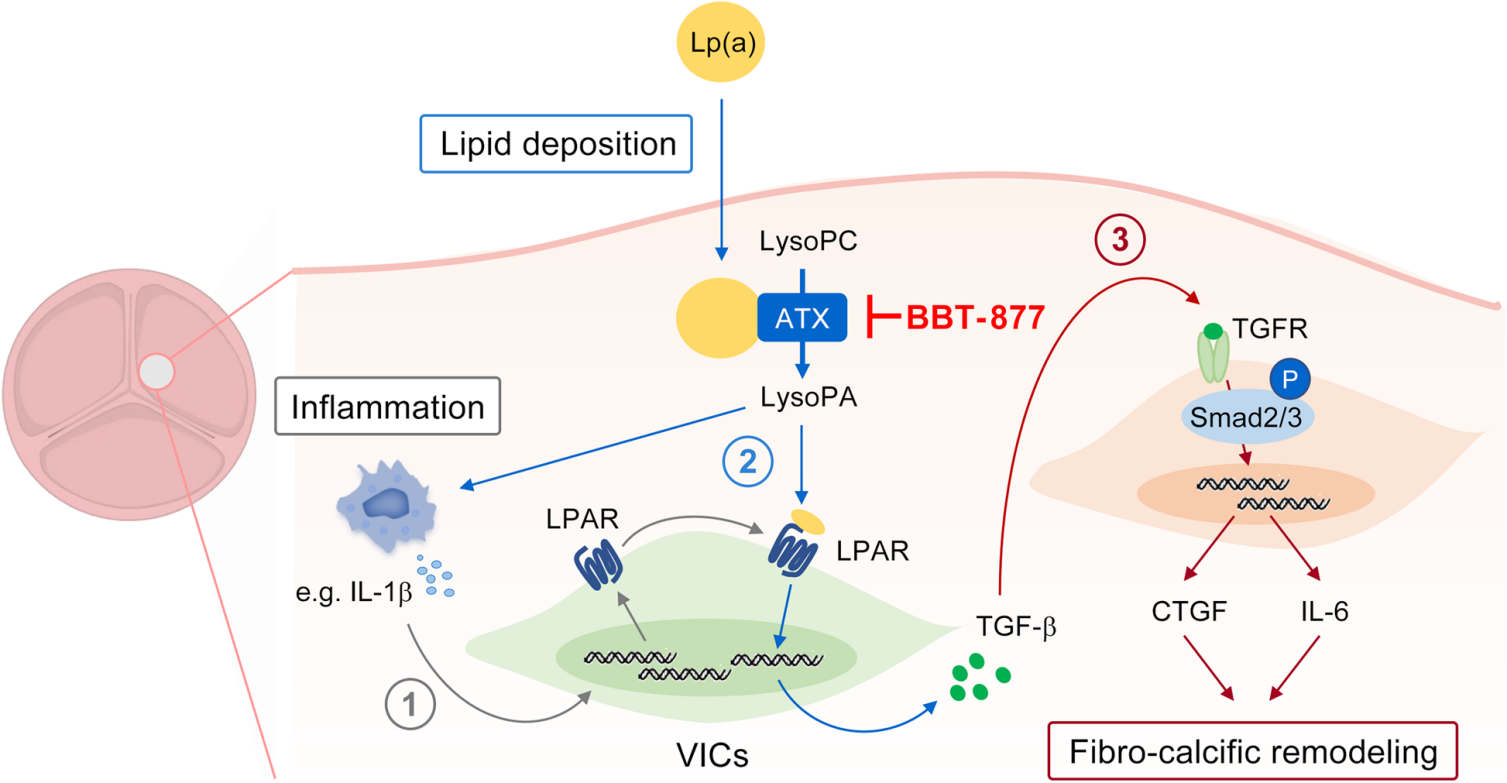
Graphical abstract. The proposed mechanism by which ATX inhibition regulates the pathological development of FCAVD is illustrated in the schematic diagram. In diseased valves, an inflammatory environment emerges, causing immune cells such as macrophages to infiltrate the valvular tissue and release inflammatory cytokines (e.g., IL-1β). ① This IL-1β upregulates the expression of LPAR1, which strengthens the action of ATX-LPA. In addition, Lp(a) carrying ATX and LPC accumulates in the valves, while activated VICs secrete additional ATX [12, 31]. ② ATX subsequently converts LPC into LPA, which binds to the increased LPA receptor (LPAR), activating the TGF-β signaling pathway [32]. ③ In turn, this activation promotes fibro-calcific remodeling by inducing the expression of CTGF and IL-6 via the TGF-β signaling axis. Importantly, ATX inhibition specifically hinders the activation of ATX-LPA enhancing TGF-β-linked signaling and ultimately alleviating fibrosis and calcium deposition in the activated VICs

We observed in our present study that ATX activity is effectively suppressed by BBT-877, a small molecule ATX inhibitor, in human VICs of patients with severe FCAVD, and that this ATX inhibition decreases the expression of genes related to both fibrosis and calcification in VICs (Fig. 7). We further found that BBT-877 is protective against aortic valve calcification, resulting in the prevention of FCAVD progression in both mouse and rabbit animal models. Thus, these findings reveal a new therapeutic target for FCAVD and indicates the potential clinical application of ATX inhibitors in affected patients.

Despite the failure of statins to target low-density lipoprotein (LDL) cholesterol and thereby prevent the progression of FCAVD, the development of alternate lipid-lowering drugs remains an attractive but still elusive endeavor. Apo B-containing atherogenic lipoprotein particles are involved in the earliest steps of aortic valve calcification and include very-low-density, intermediate-density lipoprotein particles, and lipoprotein(a) (Lp[a]) [33, 34]. Among these, Lp(a) has emerged as an attractive target, since genetic variation in the Lp(a) locus is associated with aortic valve calcification across multiple ethnic groups and with incident clinical AS [35]. In addition, patients with elevated Lp(a) levels also demonstrate faster disease progression [36] and, in an exploratory analysis of clinical trials of proprotein convertase subtilisin/kexin type 9 (PCSK9) inhibition, higher Lp(a) levels, but not Lp(a)-corrected LDL cholesterol levels, were found to be associated with a higher risk of subsequent AS events [37]. As PCSK9 inhibitors reduce LDL-cholesterol by 50 to 60% and Lp(a) by 20 to 30% [38–40], this raises the possibility that pharmacologic lipid-lowering therapy could offer a means to prevent or slow AS progression. Dedicated randomized clinical trials are therefore needed to test the clinical efficacy of conventional PCSK9 inhibitors or newly developed Lp(a) inhibitors [41, 42] in preventing AS progression. The inability of any statin to prevent AS progression is likely multifactorial and could be linked, at least in part, to the inefficacy of this class of drug to lower Lp(a). Mechanistic insights into how Lp(a) promotes an osteogenic program in human VICs have been extensively sought and have ultimately revealed that the ATX-LPA axis seems to be a critical pathway [14]. Our current study has revealed the beneficial effects of ATX inhibition against the progression of FCAVD in animal experiments and provided direct evidence in support of the ATX-LPA axis as an important pathophysiologic mechanism in this disease.

Lp(a) is a complex LDL-like particle carrying oxidized phospholipids (OxPLs) with a disulfide bridge between apolipoprotein A and apolipoprotein B and has been associated with atherosclerosis. ATX is transported in the bloodstream by Lp(a), with VICs releasing a high quantity of this protein. ATX promotes a sustained conversion of lysophosphatidyl choline to LPA, which acts as a key promotor of inflammation and mineralization of the aortic valve through a nuclear factor-kappa B (NF-κB)/interleukin-6 (IL-6)/bone morphogenetic protein (BMP) pathway, confirmed in animal experiments [43]. Since LPA can also be produced by the action of phospholipases on the membrane microvesicles shed by activated platelets [44] and platelet-derived adenosine diphosphate induces the release of ATX by VICs, an association between activated platelets and the ATX-LPA axis has been suggested as a mechanism underlying the development and progression of FCAVD [45]. In a prior cross-sectional study conducted in 300 patients with coronary artery disease, the subjects with a higher ATX activity and Lp(a) level had an elevated risk of AS. Parallel increases in ATX activity [31] and OxPLs have been confirmed in human aortic valve tissues with different pathologic grades of AS [46]. These findings collectively suggest a strong association between the ATX-LPA axis and the development of AS. In this regard, our present findings have demonstrated a preventive effect of the ATX inhibitor on AS progression and thus lend further support to the causal relationship between the ATX-LPA axis and the development and progression of AS. More importantly, in addition to its beneficial effects in preventing calcification, we have shown that ATX inhibition via BBT-877 provides additional anti-inflammatory and anti-fibrosis effects.

Lymphocyte homing and inflammation was the first process in which the ATX-LPA axis was reported to play a major role [47]. However, the ATX-LPA axis has emerged as a novel regulator of multiple molecular pathways and has been strongly implicated in a variety of physiological and pathological processes, including pulmonary fibrosis [48, 49], neuropathic pain [50], cardiovascular disease [51], and tumor progression [52–56]. Among these phenomena, the increased ATX activity in many inflammatory and fibro-proliferative conditions and the contribution of LPA to lymphocyte homing and inflammation have generated particular interest among clinicians. Furthermore, the promotion of steady-state T lymphocyte recirculation by LPA supports the idea that the ATX-LPA axis may play a broader role in inflammation. This ATX-LPA axis also amplifies cytokine production in macrophages and promotes lymphocyte infiltration, exacerbating inflammation in conditions such as pulmonary fibrosis, acute colitis, and rheumatoid arthritis [57–59]. This observation aligns with our recent findings, which indicate that LPAR1 is exclusively expressed and subsequently reinforces the ATX-LPA axis in activated VICs in response to IL-1β (Additional file 1: Fig. S5). In addition, LPA engagement has been shown to activate TGF-β signaling along with the stimulation of fibroblast accumulation, resulting in pulmonary fibrosis [60]. Given the involvement of ATX-LPA receptor signaling in several disorders characterized by inflammation and fibrosis, it is understandable that much effort has been spent in developing specific ATX inhibitors, both in academia and in industry. Idiopathic pulmonary fibrosis is the first emerging target disease for ATX inhibition as genetic and pharmacologic targeting of the ATX-LPA axis has been shown to attenuate disease development in animal experiments, which has prompted clinical trials of ATX inhibitors against idiopathic pulmonary fibrosis [15, 61].

Our study had some limitations. First, although our present in vivo observations in mice and rabbits were consistent with our data using VICs from patients with FCAVD, the pathogenetic molecular mechanisms of FCAVD in these animal models may well be different from those in humans with this disorder, and the clinical efficacy of ATX inhibitors needs to be further tested. Second, only one ATX inhibitor, BBT-877, was tested in our present experiments, and the potential impact of differences between the organ or tissue distribution of different ATX inhibitors could not therefore be evaluated. Finally, more specific LPA receptor antagonists are being actively developed and are currently available for clinical trials for patients with idiopathic pulmonary fibrosis [62, 63]. Although six different LPA receptors have been reported to be present on the surface of a wide variety of cells, additional information is needed to define the clinical role of specific LPA receptor antagonists. Head-to-head comparisons between ATX inhibitors and LPA receptor antagonists to prevent the development and progression of FCAVD are warranted.

The pleiotropic effects of ATX-mediated LPA production in human VICs, including inflammation, fibrosis, and calcification, are unique and very attractive targets for novel FCAVD therapies as ATX inhibition can potentially provide favorable effects targeting all three critical molecular pathways (inflammation, fibrosis, and calcification) associated with the development and progression of this disease [64]. Considering the current lack of medical treatments for FCAVD, these findings have potential clinical implications by suggesting a novel target for this disorder via a newly described molecular mechanism and supporting the idea that ATX inhibitors have theoretical advantages over other medications by mainly preventing the calcification of the aortic valve.

## Conclusions

In summary, we have found in our present analyses that the ATX-LPA axis is a potential new therapeutic target for the treatment or prevention of FCAVD and that ATX inhibitors may play a major future role in the medical management of patients suffering from this condition. This possibility warrants further testing using randomized clinical trials.

### Supplementary Information

**Additional file 1:** **Fig. S1.** The ATX-LPA axis is elevated in the serum of FCAVD patients. **Fig. S2.** The ATX-LPA signaling axis is activated in the fibro-calcific remodeling in FCAVD patients. **Fig. S3.** The effects of BBT-877 on cell viability in VICs. **Fig. S4.** TGF-β induced-osteogenic differentiation was diminished through ATX inhibition. **Fig. S5.** The enhanced ATX-LPA axis in VICs by inflammatory stimulation.

**Additional file 2.** The ARRIVE guidelines 2.0: author checklist.

**Additional file 3.** Original blot images.

## Data Availability

The datasets used and/or analyzed during the current study are available from the corresponding author upon reasonable request.

## Abbreviations

ALP: Alkaline phosphatase
AR: Alizarin red
AS: Aortic stenosis
*a*-SMA: Alpha-smooth muscle actin
ATX: Autotaxin
AVA: Aortic valve area
BBT: BBT-877
bFGF: Basic fibroblast growth factor
BMP2: Bone morphogenetic protein 2
Chol: Cholesterol-enriched chow
CTGF: Connective tissue growth factor
DMEM: Dulbecco’s modified Eagle’s medium
ELISA: Enzyme-linked immunosorbent assay
eNOS: Endothelial nitric oxide synthase
FBS: Fetal bovine serum
FCAVD: Fibro-calcific aortic valve disease
FLT3L: FMS-like tyrosine kinase 3 ligand
GEO: Gene expression omnibus
GM-CSF: Granulocyte-macrophage colony-stimulating factor
H&E: Hematoxylin and eosin
IFN-γ: Interferon gamma
IGFBP-3: Insulin-like growth factor binding protein 3
IHC: Immunohistochemistry
IL-1: Interleukin 1
IL-1α: Interleukin 1 alpha
IL-1β: Interleukin 1 beta
IL-4: Interleukin 4
IL-6: Interleukin 6
IL-8: Interleukin 8
IL-10: Interleukin 10
IL-12: Interleukin 12
IP-10: C-X-C motif chemokine ligand 10
LDL: Low-density lipoprotein
Lp[a]: Lipoprotein(a)
LPA: Lysophosphatidic acid
LPAR: Lysophosphatidic acid receptor
LPC: Lysophosphatidyl choline
LVOT: Left ventricular outflow tract
MCP-1: Monocyte chemoattractant protein-1
M-CSF: Macrophage-colony stimulating factor
Mean CP: Mean pressure gradient
MIF: Migration inhibitory factor
MIG: C-X-C motif chemokine ligand 9
MIP-1α: Macrophage inflammatory protein-1 alpha
MIP-1β: Macrophage inflammatory protein-1 beta
NF-κB: Nuclear factor-kappa B
NZW: New Zealand white rabbit
OxPLs: Oxidized phospholipids
PDGF-AA: Platelet-derived growth factor AA
PDGF-BB: Platelet-derived growth factor BB
PVDF: Polyvinylidene difluoride
qPCR: Quantitative real-time polymerase chain reaction
SD: Standard deviation
SDS-PAGE: Sodium dodecyl sulfate-polyacrylamide gel electrophoresis
TGF-β: Transforming growth factor beta
TNF-α: Tumor necrosis factor alpha
TRAIL: TNF-related apoptosis-inducing ligand
VECs: Valvular endothelial cells
VEGF: Vascular endothelial growth factor
VICs: Valvular interstitial cells
VitD: Vitamin D2
*V*max: Transaortic maximal velocity
