# Correction: Autotaxin inhibition attenuates the aortic valve calcification by suppressing inflammation-driven fibro-calcific remodeling of valvular interstitial cells

**DOI:** 10.1186/s12916-024-03403-1

**Published:** 2024-04-29

**Authors:** Dohee Yoon, Bongkun Choi, Ji-Eun Kim, Eun-Young Kim, Soo-Hyun Chung, Hyo-Jin Min, Yoolim Sung, Eun-Ju Chang, Jae-Kwan Song

**Affiliations:** 1 Department of Biochemistry and Molecular Biology, Brain Korea 21 Project, Asan Medical Center, University of Ulsan College of Medicine, 88 Olympic-Ro 43-Gil, Songpa-Gu, Seoul, 05505 Republic of Korea; 2 Stem Cell Immunomodulation Research Center, Asan Medical Center, University of Ulsan College of Medicine, 88 Olympic-Ro 43-Gil, Songpa-Gu, Seoul, 05505 Republic of Korea; 3 Division of Cardiology, Department of Internal Medicine, Asan Medical Center, University of Ulsan College of Medicine, 88 Olympic-Ro 43-Gil, Songpa-Gu, Seoul, 05505 Republic of Korea

**Correction** **:** **BMC Med 22, 122 (2024)**

**https://doi.org/10.1186/s12916-024-03342-x**

The authors wish to note the following Funding acknowledgement which was mistakenly omitted from the original article [1]:

‘This work is also supported by a grant from Bridge Biotherapeutics (Gyeonggi, Republic of Korea).’
