# Supplementary material for: Autotaxin inhibition attenuates the aortic valve calcification by suppressing inflammation-driven fibro-calcific remodeling of valvular interstitial cells
Source: BMC Med. 2024 Mar 14;22:122. doi: 10.1186/s12916-024-03342-x (PMC10941471; doi:10.1186/s12916-024-03342-x)
Supplement: Supplementary file 1 — Additional file 1: Fig. S1. The ATX-LPA axis is elevated in the serum of FCAVD patients. Fig. S2. The ATX-LPA signaling axis is activated in the fibro-calcific remodeling in FCAVD patients. Fig. S3. The effects of BBT-877 on cell viability in VICs. Fig. S4. TGF-β induced-osteogenic differentiation was diminished through ATX inhibition. Fig. S5. The enhanced ATX-LPA axis in VICs by inflammatory stimulation. [file 12916_2024_3342_MOESM1_ESM.docx]

**SUPPLEMENTARY INFORMATION**

**S1. SUPPLEMENTARY LEGENDS**


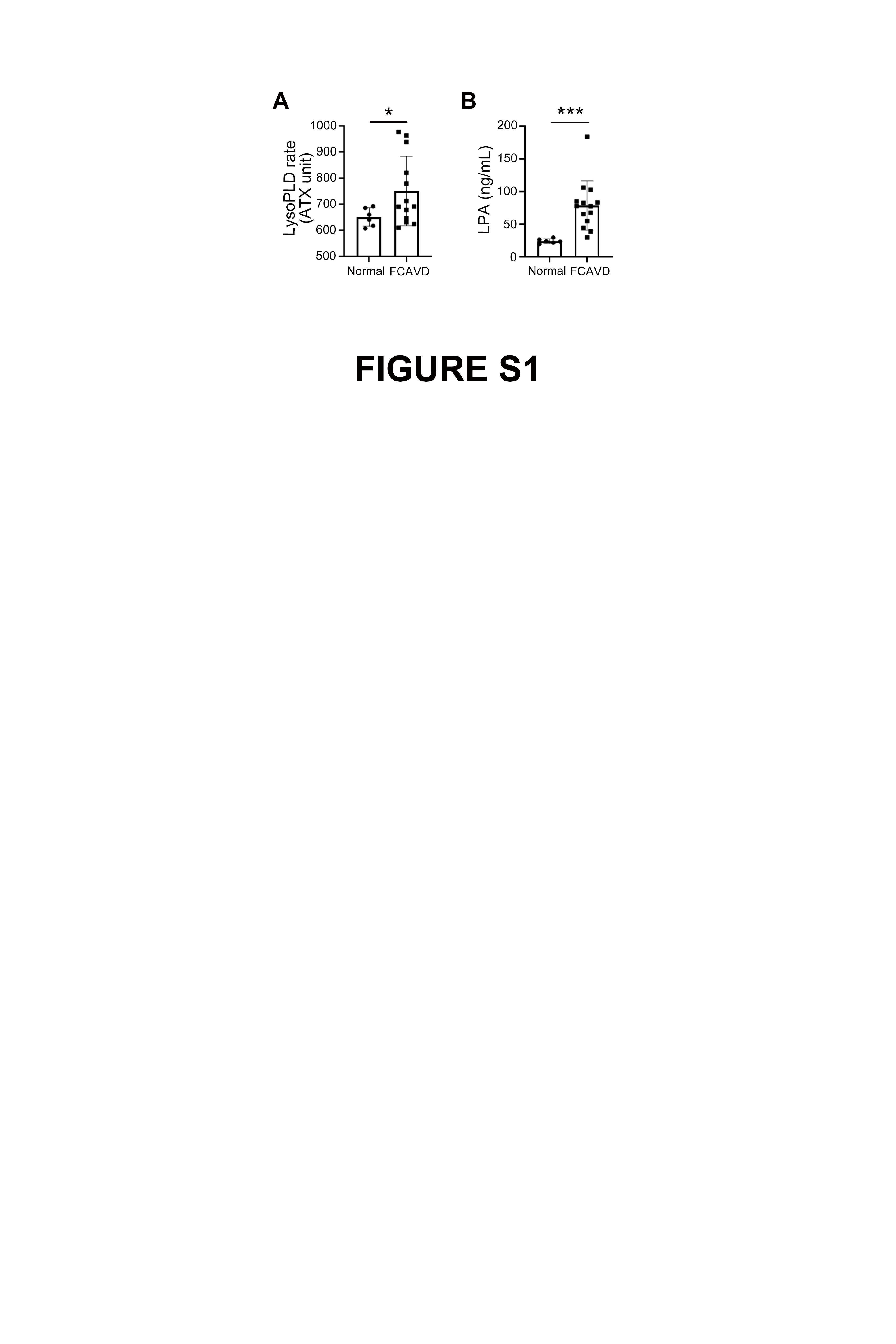


**Fig. S1** The ATX-LPA axis is elevated in the serum of FCAVD patients. **A**, **B** ATX activity **A** and LPA production **B** in serum were measured using ELISA kits in healthy (n=6) and FCAVD patients (n=14). Data are presented as the mean ± SD. **P*<0.05, *** *P* <0.001 versus the normal sample. P values were obtained using a two-tailed t-test.


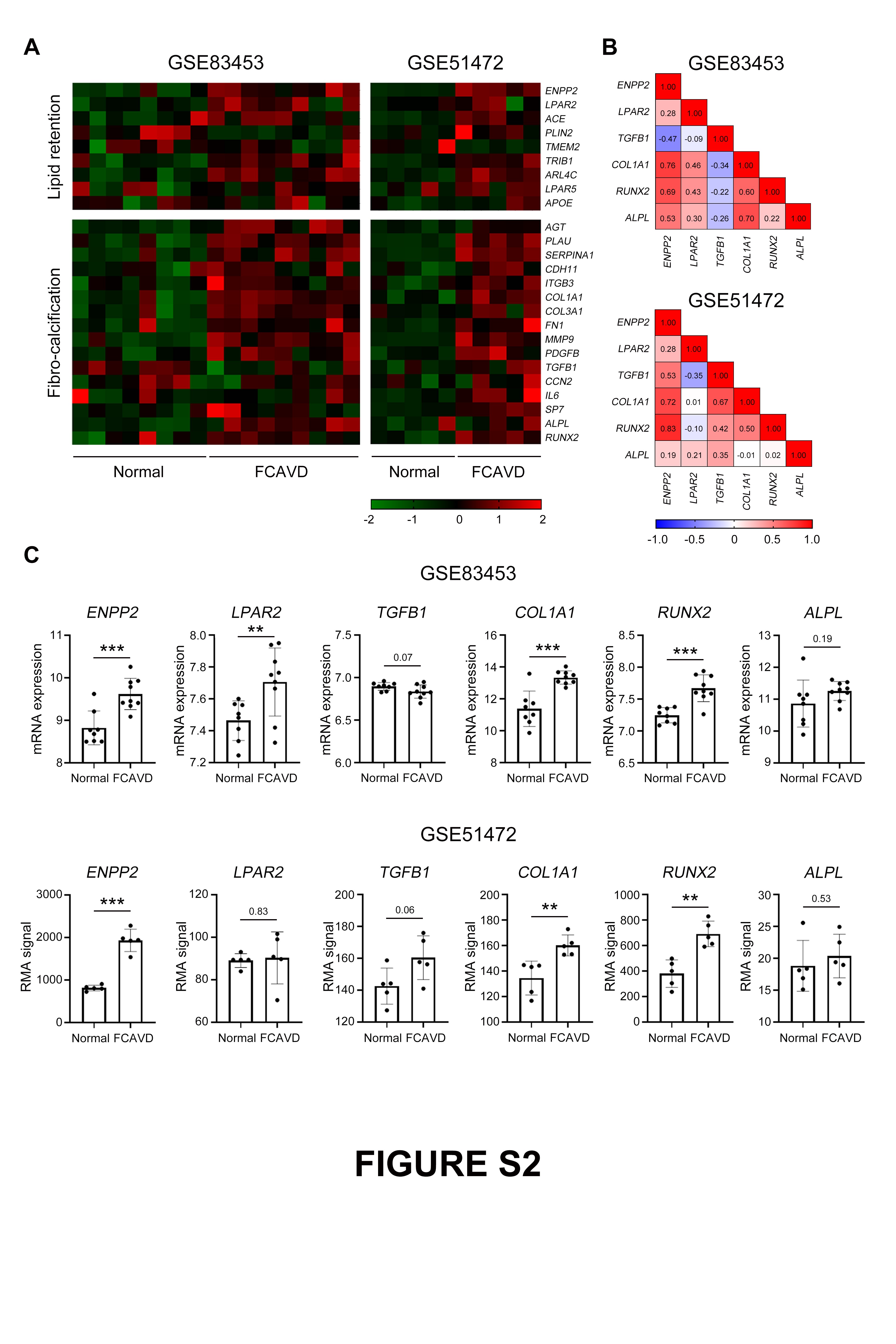


**Fig. S2** The ATX-LPA signaling axis is activated in the fibro-calcific remodeling in FCAVD patients. **A-C** Related genes of lipid retention and fibro-calcific changes were compared between healthy and FCAVD patients. **A** Data were obtained from the GSE83453 and GSE51472 datasets and visualized using MeV 4.9.0 heatmap software. **B** Pearson correlation values of genes associated with lipid retention and fibro-calcific changes were analyzed to examine the correlation between ATX and selected fibro-calcific-related genes. **C** The mRNA expressions of genes associated with lipid retention and fibro-calcific changes were compared between aortic valvular tissue of patients with FCAVD and age-matched non-FCAVD controls using a two-tailed t-test. **P*<0.05, *** *P*<0.01, *** *P*<0.001 versus the healthy sample. Data are presented as the mean ± SD. (GSE83453; n=8 for healthy and n=9 for FCAVD patients, GSE51472; n=5 per group)

­­


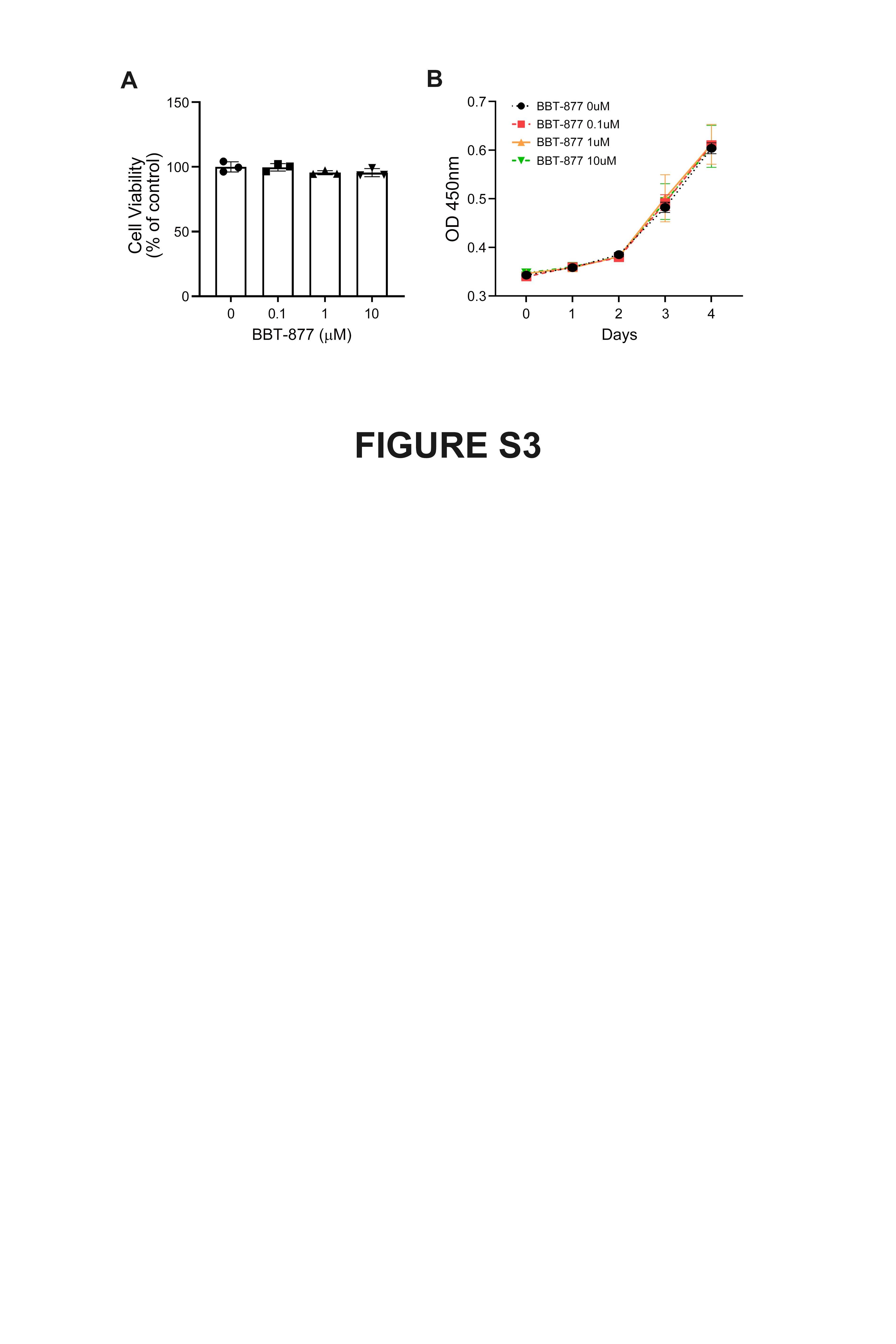


**Fig. S3** The effects of BBT-877 on cell viability in VICs. **A, B** Cell viability **A** and proliferation curve **B** of VICs during various concentrations of BBT-877 treatments (0.1, 1, 10 μM). Data are presented as the mean ± SD. The experiments were performed independently in triplicate. P values were obtained using a two-tailed t-test.


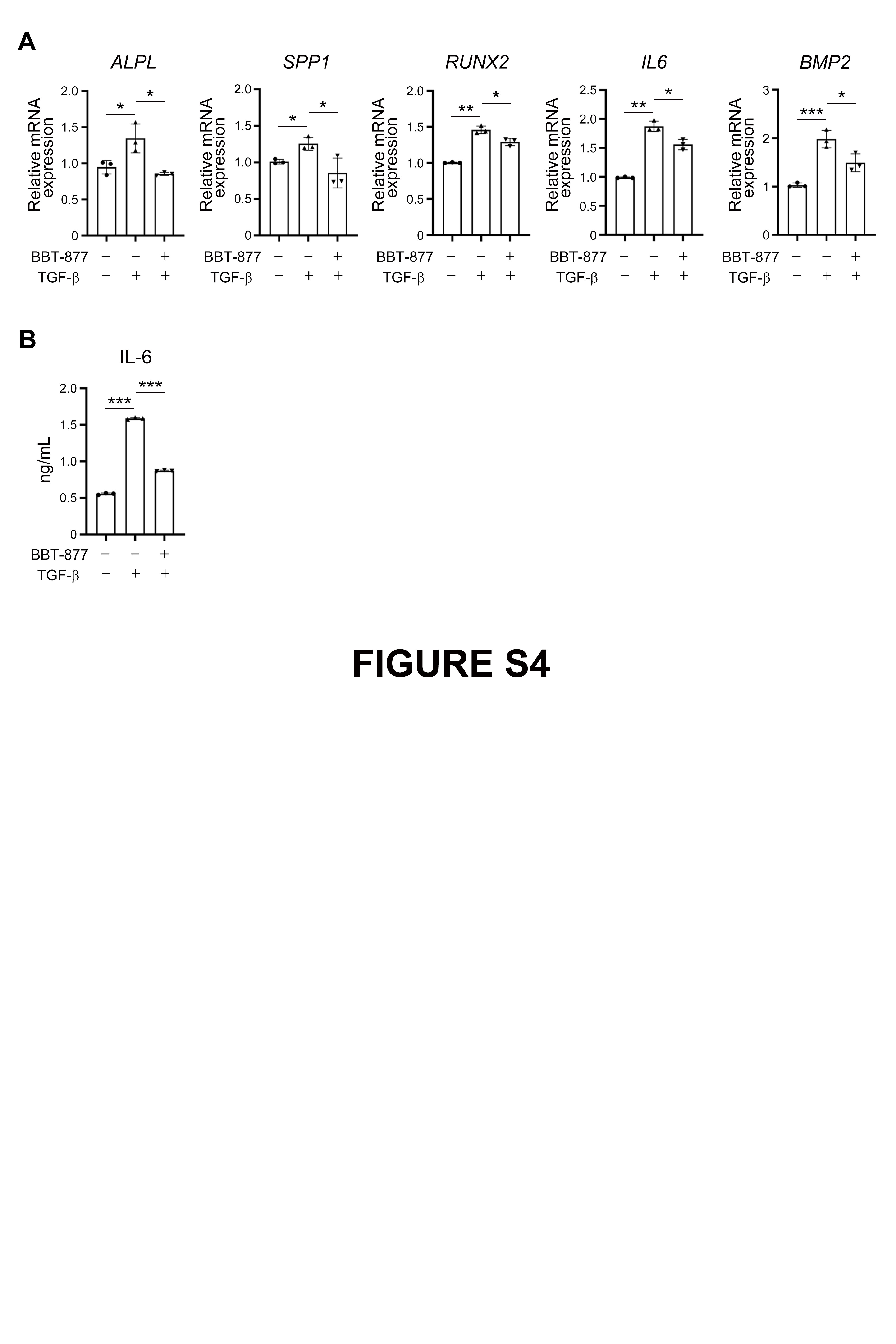


**Fig. S4** TGF-β induced-osteogenic differentiation was diminished through ATX inhibition. **A** The mRNA expression levels of *ALPL, SPP1, RUNX2, IL6,* and *BMP2* in the VICs treated with TGF-β (5 ng/mL) for 24 h in the presence or absence of BBT-877 (1 μM). **B** The protein levels of IL-6 in VICs-conditioned media after 24 h of TGF-β (5 ng/mL) stimulation in the presence or absence of BBT-877 (1 μM). Data are presented as the mean ± SD. The experiments were performed independently in triplicate. **P*<0.05, ** *P* <0.01, *** *P* <0.001 versus the indicated group. P values were obtained using a two-tailed t-test.


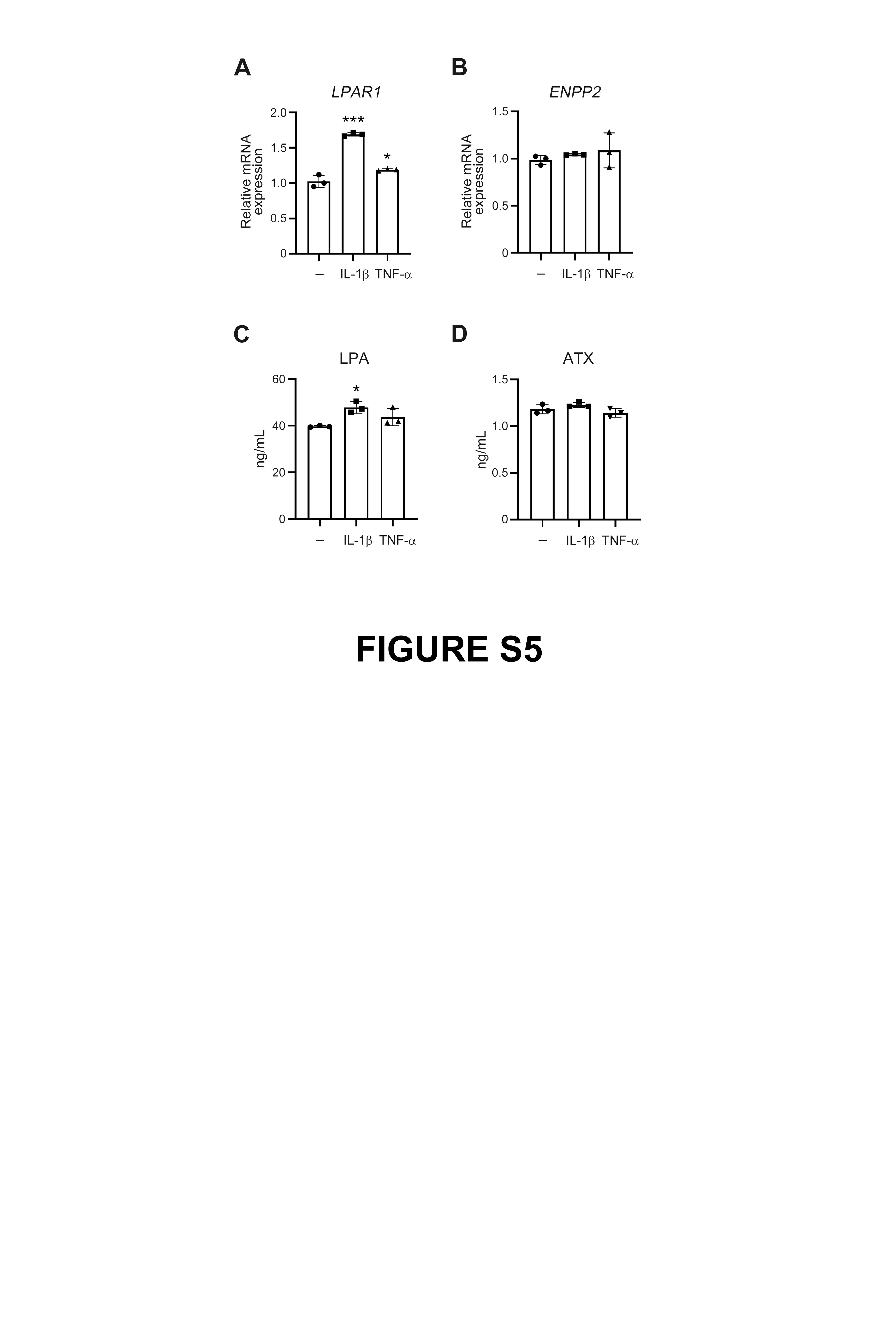


**Fig. S5** The enhanced ATX-LPA axis in VICs by inflammatory stimulation. VICs were stimulated with IL-1β (20 ng/mL) or TNF-α (50 ng/mL) for 24 h. **A, B** The mRNA expression levels of LPA receptor 1 (*LPAR1*) **A** and ATX (*ENPP2*) **B** in VICs. **C, D** The protein levels of LPA **C** and ATX **D** from the conditioned medium of VICs. Data are presented as the mean ± SD. The experiments were performed independently in triplicate. **P*<0.05, *** *P* <0.001 versus the vehicle control. P values were obtained using a two-tailed t-test.
