# Supplementary figures and images for: Autotaxin inhibition attenuates the aortic valve calcification by suppressing inflammation-driven fibro-calcific remodeling of valvular interstitial cells
Source: BMC Med. 2024 Mar 14;22:122. doi: 10.1186/s12916-024-03342-x (PMC10941471; doi:10.1186/s12916-024-03342-x)

**Figure 4A. Original blot images**


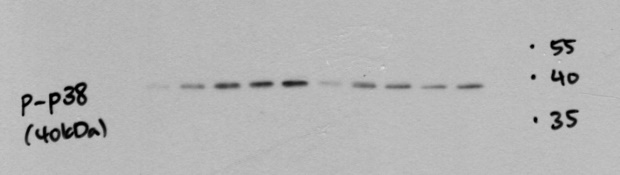

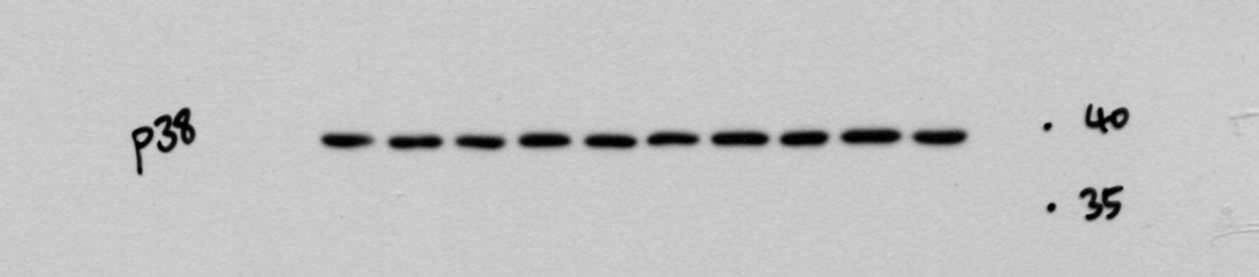

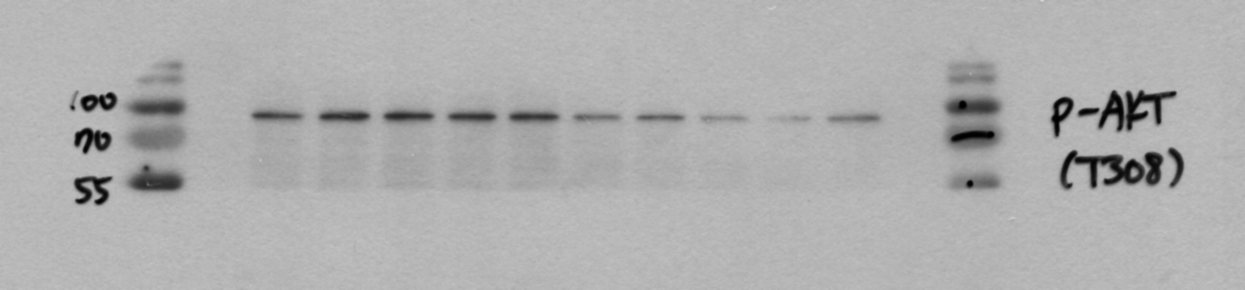

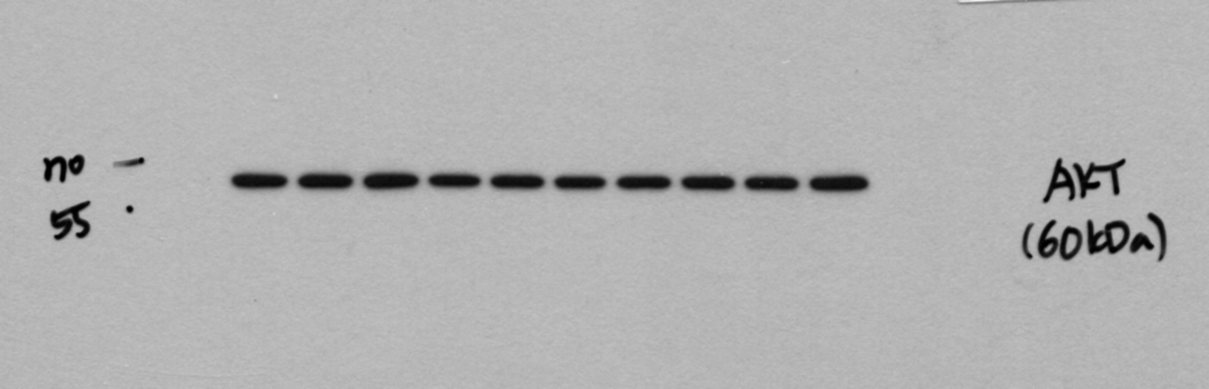

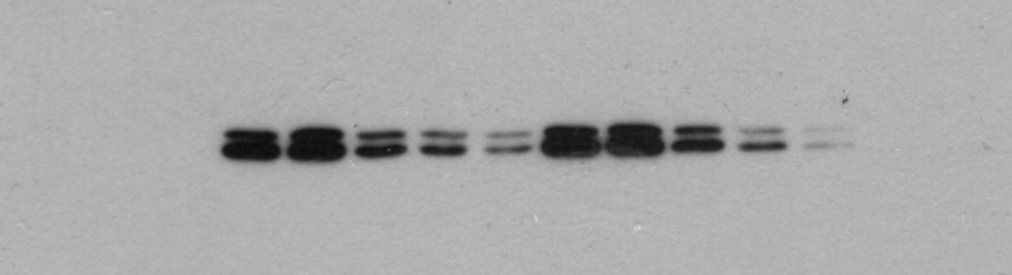


p-ERK


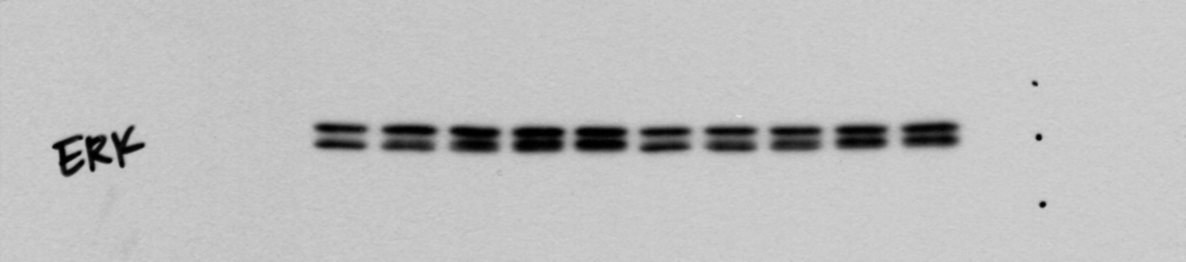

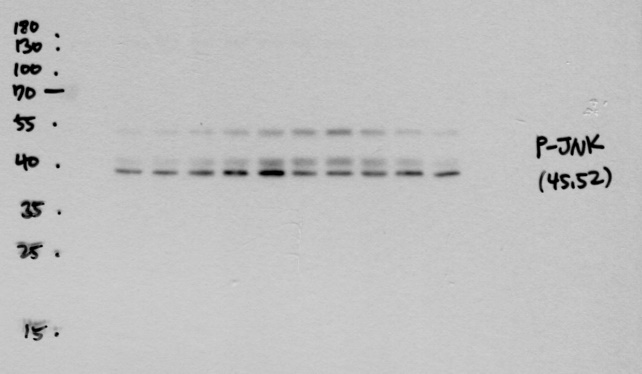

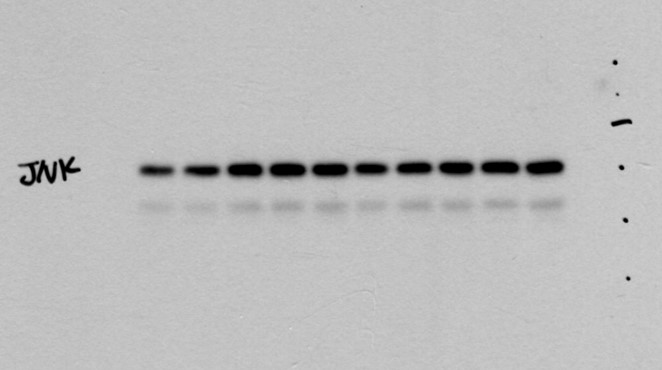

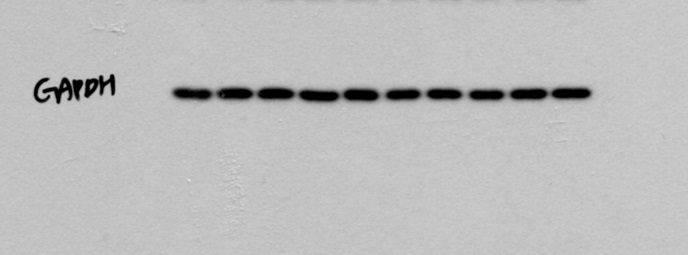

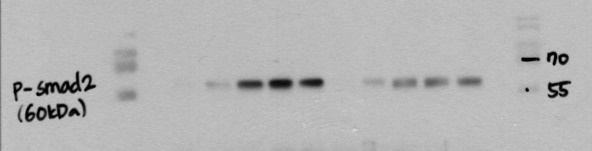

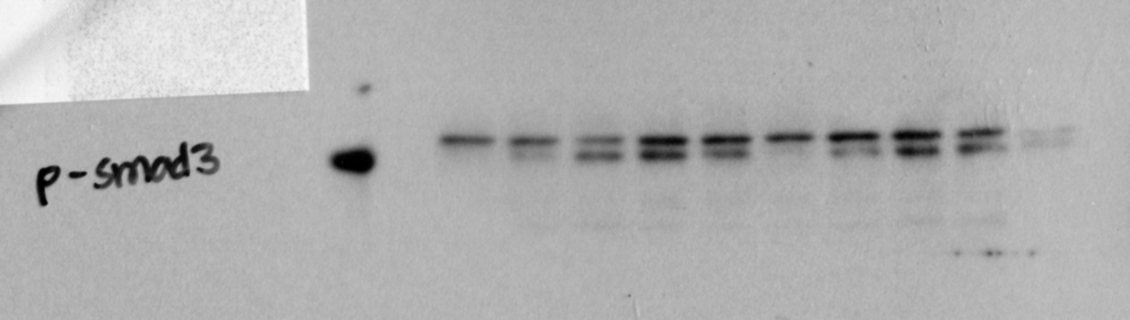

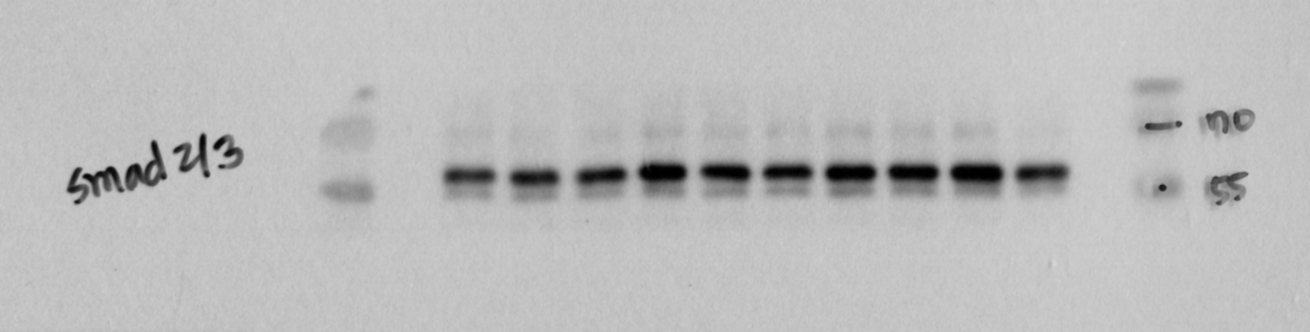

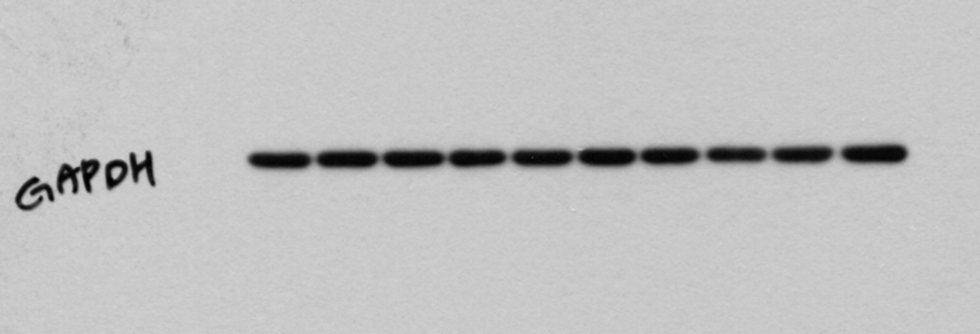


**Figure 4C. Original blot images**

Supplement: Supplementary file 3 — Additional file 3. Original blot images. [file 12916_2024_3342_MOESM3_ESM.docx]
